# An Improved Approach to Identify Bacterial Pathogens to Human in Environmental Metagenome

**DOI:** 10.4014/jmb.2005.05033

**Published:** 2020-06-18

**Authors:** Jihoon Yang, Adina Howe, Jaejin Lee, Keunje Yoo, Joonhong Park

**Affiliations:** 1 Department of Civil and Environmental Engineering, Yonsei University, Seoul 03722, Republic of Korea; 2 Department of Agricultural and Biosystems Engineering, Iowa State University, Ames, IA 50011, USA; 3 Department of Environmental Engineering, Korea Maritime and Ocean University, Busan 49112, Republic of Korea

**Keywords:** Environmental metagenome, bacterial pathogens, mtagenomic pathogen identification, microbial risk assessment

## Abstract

The identification of bacterial pathogens to humans is critical for environmental microbial risk assessment. However, current methods for identifying pathogens in environmental samples are limited in their ability to detect highly diverse bacterial communities and accurately differentiate pathogens from commensal bacteria. In the present study, we suggest an improved approach using a combination of identification results obtained from multiple databases, including the multilocus sequence typing (MLST) database, virulence factor database (VFDB), and pathosystems resource integration center (PATRIC) databases to resolve current challenges. By integrating the identification results from multiple databases, potential bacterial pathogens in metagenomes were identified and classified into eight different groups. Based on the distribution of genes in each group, we proposed an equation to calculate the metagenomic pathogen identification index (MPII) of each metagenome based on the weighted abundance of identified sequences in each database. We found that the accuracy of pathogen identification was improved by using combinations of multiple databases compared to that of individual databases. When the approach was applied to environmental metagenomes, metagenomes associated with activated sludge were estimated with higher MPII than other environments (*i.e*., drinking water, ocean water, ocean sediment, and freshwater sediment). The calculated MPII values were statistically distinguishable among different environments (*p* < 0.05). These results demonstrate that the suggested approach allows more for more accurate identification of the pathogens associated with metagenomes.

## Introduction

Bacterial pathogens and their association with diseases have long been major public health concerns around the world. It is estimated that 10 million people died of infectious diseases in 2016 [1, 2]. Our ability to identify and track these pathogens in the environment is important to understand their risks in the environment and our lives [3, 4]. Several approaches have been used to detect and identify pathogens in the environment. The culture- dependent method, which selectively grows and isolates cultivable pathogens, has been considered as a standard methodology. The culture-dependent method has the advantage of widely accessible and cost-effective [5]. The challenge of this approach is that many pathogens in the environment exist in a viable but non-culturable (VBNC) state, which leads to limited detection and underestimation of total pathogens in the environmental samples [6, 7]. To circumvent these drawbacks, molecular biological tools such as real-time quantitative PCR (qPCR) and sequencing-based approaches have been adopted to detect and identify the bacterial pathogens in the environment [8-12]. qPCR is a highly sensitive and specific method to reliably quantify the pathogenic genes in the environment using gene probes designed to target specific genes of interest. qPCR method relies on the specificity and sensitivity of primer sets to amplify the target genes. Consequently, when the number of target genes is large or unspecified, this approach is not always appropriate to identify genes associated with pathogens in the environment due to both practical and economic reasons [13, 14].

Recently, shotgun high-throughput sequencing of metagenomes from environmental microbial communities has been applied to identify bacterial pathogens in diverse environments [11, 12]. Several curated databases and tools, including the Meta-multilocus sequence typing (MLST), virulence factor database (VFDB), and pathosystems resource integration center (PATRIC), have been developed to annotate pathogens in metagenomes [15-17]. Each database is used to identify bacterial pathogens based on sequence homology to genes associated with pathogens, such as housekeeping genes in known pathogens (*i.e*., MLST) or sequence similarity with virulence genes (*i.e*., VFDB) or genome sequences of human-specific pathogens (*i.e*., PATRIC) [4,18-22]. However, relying solely on a single database to identify pathogens may have disadvantages. For example, the MLST database only contains sequence information obtained from cultivated pathogens within a relatively low phylogenetic diversity [23] and thus is limited to detecting diverse pathogens. This database can be especially challenged to differentiate pathogens when there are many clonal complexes of pathogens in an environmental metagenome [23, 25]. This limitation can be improved by using specific virulence genes for detection, like genes that are available in the VFDB. But this database has been shown to have a high occurrence of false positives errors when annotating pathogens [26-28]. The PATRIC database can be an alternative database to annotate pathogens and includes a larger amount of genomic information compared to MLST database and VFDB; however, curation of the PATRIC database is relatively poor [29, 30], and it only accepts assembled contigs in its current annotation workflow [31].

Given the limitations of each individual database for annotating pathogens, we hypothesize that utilizing a combination of identification results of all three databases would offset the drawbacks of each individual database and perform more accurate pathogen annotation. In this study, we used artificial metagenomes to compare the accuracy of pathogen identification between single databases (*i.e*., the MLST database, VFDB, and PATRIC database) and our suggested approach. In addition, a quantitative index, which summarizes qualitative information obtained from multiple databases, can be helpful to convey a comprehensive understanding of the complex pathogen identification results [32]. Thus, we propose a quantitative index to describe the degree of pathogen association within a metagenome, which is based on information obtained from multiple annotation databases. Additionally, we compare the estimated indices of pathogen association between varying environmental metagenomes.

## Materials and Methods

### Collecting and Customizing the Pathogen Databases

The MLST database (https://pubmlst.org/data/, accessed on 02/15/2019) was downloaded and contained the 251,429 sequences of 132 pathogenic species. The VFDB included 32,522 sequences of virulence genes that originated from 262 pathogenic species (http://www.mgc.ac.cn/cgi-bin/VFs/, accessed on 02/18/2019). The PATRIC database (https://www.patricbrc.org/, accessed on 03/08/2019) consisted of 146,883 sequences that originated from 1,013 pathogenic species.

We customized the information obtained from the databases to improve the accuracy of pathogen identification. First, all archaeal genes were removed from the MLST database resulting in 248,240 sequences of 111 pathogenic species. In the case of the VFDB, 32,312 sequences of 184 pathogenic species remained after removing hypothetical virulence proteins and sequences which had insufficient information (*i.e*., incomplete information on virulence factors, such as full name, structure, function, and mechanism). For the PATRIC database, non-human pathogens were excluded, and 63,846 sequences of 344 pathogenic species remained.

### Constructing the Artificial Metagenome Datasets

A total of 555 artificial metagenome datasets were constructed using combinations of 50 pathogen and 50 nonpathogen genomes. These artificial metagenomes were used to assess the ability to identify pathogens using a single database and our integrated approach (Table S1). The 50 pathogens were selected from verified pathogens in the MLST database, VFDB, PATRIC database, and the National Institute of Allergy and Infectious Diseases (NIAID, https://www.niaid.nih.gov/research/emerging-infectious-diseases-pathogens). To investigate the effect of the phylogenetic closeness on the accuracy of identification, the genomes of nonpathogenic bacteria were also included [33-35].

A phylogenetic tree to describe membership in artificial metagenome datasets and to compare the phylogenic relationship among pathogens and nonpathogens was constructed using BioEdit version 7.2.5 [36] and MEGA X [37] with the following parameters: neighbor-joining method, the bootstrap method with 1,000 replications, and maximum composite likelihood substitution (Fig. S1).

MetaSim version 0.9.5 is a shotgun sequencing simulator which generates various combinations of metagenomic reads considering genomic profile parameters that mimic errors caused by each sequencing platform [38], Using the MetaSim, artificial metagenome datasets with different ratios of pathogenic sequences (*i.e*., 0%, 1%, 2%, 3%, 4%, 5%, 6%, 7%, 8%, 9%, 10%, 50%, 90%, and 100% pathogens) were generated through the empirical error model for the modified Illumina sequencing platform which produces synthetic substitutions, insertions, and deletions. Each artificial metagenome contained 2,000,000 reads with an average read length of 150 bp and a standard deviation of 5 bp. As the usual abundance range of pathogens in the environmental samples is between 0% and 10% [20, 39, 40], artificial metagenomes which contained 0% to 10% pathogenic sequences were prioritized (*i.e*., 552 out of 555).

### Assessing the Performance of Bacterial Pathogen Identification

A receiver operating characteristic (ROC) analysis [41] was conducted to compare the annotation of pathogens of a single database approach and that of the suggested approach. Results of pathogen detection can be classified into one of four cases: true positive (TP), false positive (FP, *i.e.*, a nonpathogenic bacteria misannotated as a pathogen), true negative (TN), and false negative (FN, *i.e.*, a pathogen misannotated as a nonpathogen). We estimated the specificity (the true negative rate) and the sensitivity (the true positive rate) for each approach. Sensitivity and specificity were calculated using the formulas (TP/(TP+FN)) and (TN/(TN+FP), respectively. The ROC curve was drawn by 1-specificity and sensitivity as x and y axis [42]. The accuracy of each approach was assessed by calculating the area under the ROC curve (AUC). A classification model can be considered as effective when the AUC value is 0.8 or more, acceptable when the value is between 0.7 and less than 0.8, and poor when it is less than 0.7 [43, 44].

### Collection of Environmental Metagenomes

To examine the applicability of the suggested approach, publicly available metagenomes from diverse environments were collected and tested. A total of 70 environmental metagenomes were collected from the MG- RAST (https://www.mg-rast.org/) and the NCBI sequence read archive (SRA; https://www.ncbi.nlm.nih.gov/sra) (Table S2). The environments of the collected metagenomes can be classified into (i) wastewater-treatment activated sludge (32 metagenomes, W1-W32, [45]); (ii) drinking water (29 metagenomes, D1-D29, [46, 47]); (iii) sediments (5 metagenomes, S1-S5, [48]); and (iv) ocean water (4 metagenomes, O1-O4, [49]). For quality control, reads with a length of less than 100 bp, which increase the annotation error rate [50, 51], were removed from environmental metagenomes.

### Bioinformatics Analysis of Metagenome Datasets

The bioinformatics strategy for metagenome datasets consisted of three steps: (i) quality filtering (described above), (ii) annotation, and (iii) weighting coefficient derivation. Each artificial metagenome was annotated against pathogen-associated genes in each customized MLST database, VFDB, and PATRIC database using standalone BLASTN (version 2.9.0). A threshold of *E*-value, representing alignment similarity and alignment length, was set to 10^-3^ to minimize misannotations [50, 52] and the aligned match with the highest similarity (*e.g*., highest bit-score) was chosen for further analysis. For pathogen identification using the MLST database, sequences associated with pathogens in metagenome datasets were considered as a positive alignment only if all the housekeeping genes corresponding to a gene profile were also found in a metagenome, as previously described [15]. For each metagenome sequence, we tracked its identification as a pathogen against each database. Each sequence associated with a pathogen could be categorized into one of eight groups depending on which database was used for alignment. Group *MVP* contained sequences annotated by all the VFDB (virulence genes), PATRIC (genomes of pathogens), and MLST database (housekeeping genes). Groups *MV*, *MP*, and *VP* contained hits from two of the three databases. The sequences detected by a single database belonged to Groups *M*, *V*, and *P*. Group *None* was a collection that was not detected by any database (Fig. S2).

Alignment to specific or multiple database genes could result in more confidence in pathogen identification. For a brief and comprehensive understanding of the identification results, we developed the “Metagenomic Pathogen Identification Index” (MPII). Metagenomic pathogen identification indicates a broad approach to search gene sequences or fragments of infectious pathogens within deep-sequenced datasets [14]. The definition of MPII is an index describing the degree of pathogen association within a metagenome. If a sequence was detected by multiple pathogen associated databases, higher weighting coefficients were assigned to the sequence based on its classified group. To derive the weighting coefficient of each group, a multiple linear regression (MLR) model was applied [53]. The proportion of associated pathogen sequences (*i.e*., 0%, 1%, 2%, etc.) in a metagenome and the relative abundance of each group (*i.e*., the relative abundance of group *MVP*, *MV*, and *VP*, etc. in the metagenome) were used as variables. Generally, MLR describes the relationship between a dependent variable and several independent variables. In a MLR analysis, the error term denoted by ε is assumed to be normally distributed with mean 0 and variance σ^2^ (which is a constant). In our analysis, ε is also assumed to be uncorrelated. Thus, the regression equation can be written as:

(1) $$y=b_{0}+\sum_{i=1}^{n}b_{i}x_{i}+\varepsilon$$

where b_i_ are the regression coefficients, xi are independent variables, and ε is the stochastic error associated with the regression.

The least squares method was used for the derivation of coefficients. The variables having strong collinearity were excluded from the analysis, and a stepwise regression procedure was employed to select the independent variables that would result in the optimal equation. For training the model, 70% of the entire dataset was used, and the remaining 30% dataset was used to validate MLR model performance [54]. The root mean square error (RMSE), coefficient of determination (R^2^), and adjusted coefficient of determination (Adjusted R^2^) were used to evaluate the performance of MLR model according to a previous study [55]. Multicollinearity assumption was verified by Variable of Inflation (VIF) accompanied by the MLR output. When the average VIF is under 10, the conducted regression considered acceptable [56, 57]. The MLR analysis was conducted using the SAS program, version 9.4 (SAS Institute Inc., USA). A non-parametric Kruskal–Wallis H test and post hoc Dunn’s test was conducted to identify statistically significant differences among the MPII of environmental metagenomes. The 5% significance level was adopted.

## Results

### Improved Pathogen Detection Obtained by Using the Combination of Three Customized Databases

Pathogenic sequences within the artificial metagenomes were annotated against each of the three customized databases, and the identification results were integrated for further analysis (Fig. S3). Using the MLST database alone, 37 out of 50 pathogens were correctly identified. There were no nonpathogens annotated as a pathogen (*i.e*., no false positives). *Mycobacterium*, and *Campylobacter,* and two of three species of *Clostridium* and *Burkholderia* genera were not detected although the MLST database contained the housekeeping gene of those species. *Legionella*, *Rickettsia*, *Shigella*, *Francisella*, *Coxiella* were not annotated as pathogens because there was no genetic information of those genera in the MLST database. The VFDB annotated 46 out of 50 pathogens, while two nonpathogens misannotated as pathogens. The nonpathogenic *Brevibacillus,* phylogenetically close to the pathogenic *Bacillus* and *Clostridium* species, was annotated as a pathogen by VFDB. The pathogenic species of *Chlamydia*, *Campylobacter, Borrelia,* and *Ureaplasma* were not detected by VFDB. Among those genera, the virulence gene sequences of *Chlamydia* and *Campylobacter* species were included in the VFDB. The PATRIC database identified 41 pathogens from the artificial metagenomes with one false positive (nonpathogenic *Bacillus* species). *Borrelia, Clostridioides, Haemophilus, Legionella, Bordetella, Aeromonas, Neisseria, Ureaplasma*, and *Anaplasma* were not detected by PATRIC database. We observed that the proportion of pathogens in artificial metagenomes did not significantly affect pathogen detection. Among the databases, VFDB showed the highest sensitivity (0.91), followed by the PATRIC database (0.82) and the MLST database (0.74). In terms of specificity, all three databases showed high values (over 0.97). Overall, the VFDB (0.95) and the PATRIC database (0.90) were more accurate than the MLST database to identify pathogens in the artificial metagenomes (Table 1).

Pathogen identification using our suggested approach (*i.e*., integrating the identification results from the three databases) correctly identified 48 out of 50 pathogens (Fig. S3). The one nonpathogenic *Bacillus,* phylogenetically close to other pathogenic *Bacillus* species, was annotated as a pathogen. Compared to the single database approach, the suggested approach significantly improved sensitivity (0.96) and showed the highest accuracy (0.97). These results also demonstrated that the suggested approach resulted in the fewest false negative identification of pathogens.

### Derivation of an Equation to Calculate Metagenomic Pathogen Identification Index

As the abundance of pathogenic sequences increased in artificial metagenomes, the number of metagenomic reads annotated as a pathogen increased. As annotations were classified into groups identifying their association with specific databases, we observed increases in all groups except for Group *MV* and Group *None* (Table S3). Generally, the abundance of pathogens in the artificial metagenomes (*i.e*., pathogenic sequence ratio ranges between 0% to 100%) was linearly correlated with the relative abundance of the identified pathogenic sequences in each group.

Unlike other groups, the regression coefficient of Group *MV* could not be obtained because no observed artificial sequence was annotated and assigned to Group *MV* (Table 2). Thus, Group *MV* was excluded from deriving an equation of the presence of the verified pathogen based on annotation characteristics. Based on the MLR analysis, we derived Eq. (2) to calculate the MPII value of the metagenomes.

(2) $$Metagenomic\;Pathogen\;Identification\;Index\;\left(MPII\right)=\;821.95\;\times \;G_{MVP}+\;1.92\;\times \;G_{VP}+\;12.14\;\times \;G_{V}–\;63.20\;\times \;G_{MP}+\;1.37\;\times \;G_{P}+\;292.66\;\times \;G_{M}-\;2.206$$

where G_i_ is the relative abundance (%) of each group in a single metagenome.

The performance evaluation of the MLR analysis confirmed that the derived regression equation was acceptable. The R^2^ and Adjusted R^2^ values ranged between 0.72 and 0.81 for both the training and the test set. RMSE value for the training and the test set were 8.39 and 11.28, respectively, which were within acceptable ranges. Multicollinearity between independent variables was also checked using VIF, and no multicollinearity was found (Table S4).

**Fig. 1:**
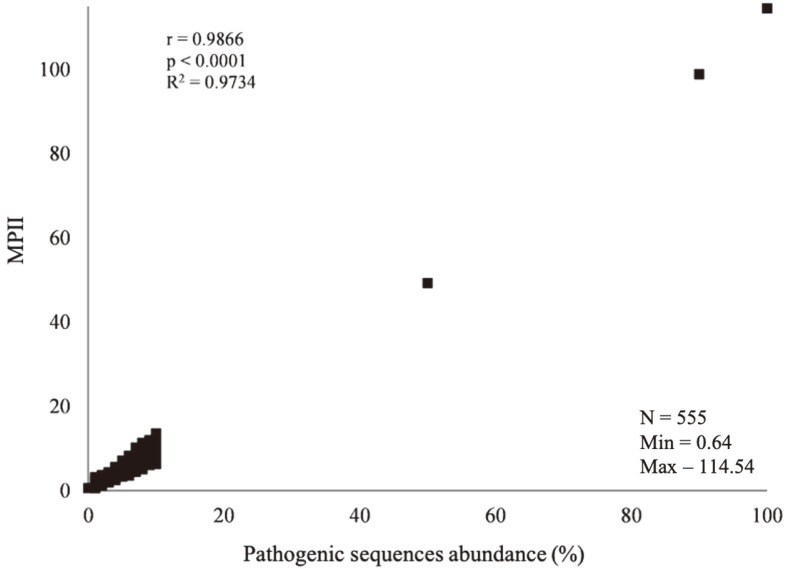
Calculated MPII values of artificial metagenomes that contain various ratios of pathogenic sequences. The MPII values of 555 artificial metagenomes were calculated using the equation derived from the multiple linear regression (MLR) analysis. The relative abundances of the categorized groups for each metagenome were used to calculate the MPII values. The correlation coefficient (r), *p*-value (*p*), and coefficient of determination (R^2^) were calculated using Pearson linear regression analysis.

The MPII calculated from the artificial metagenome containing only nonpathogenic sequences (*i.e*., no pathogenic sequences in the metagenome) was 0.64, and the value for the artificial metagenome containing only pathogenic sequences was 114.54 (Fig. 1). The significant positive relationship was shown between the abundance of pathogen and MPII (r = 0.9866, *p* < 0.0001).

### Applicability of the Derived Equation for Environmental Metagenomes

**Fig. 2:**
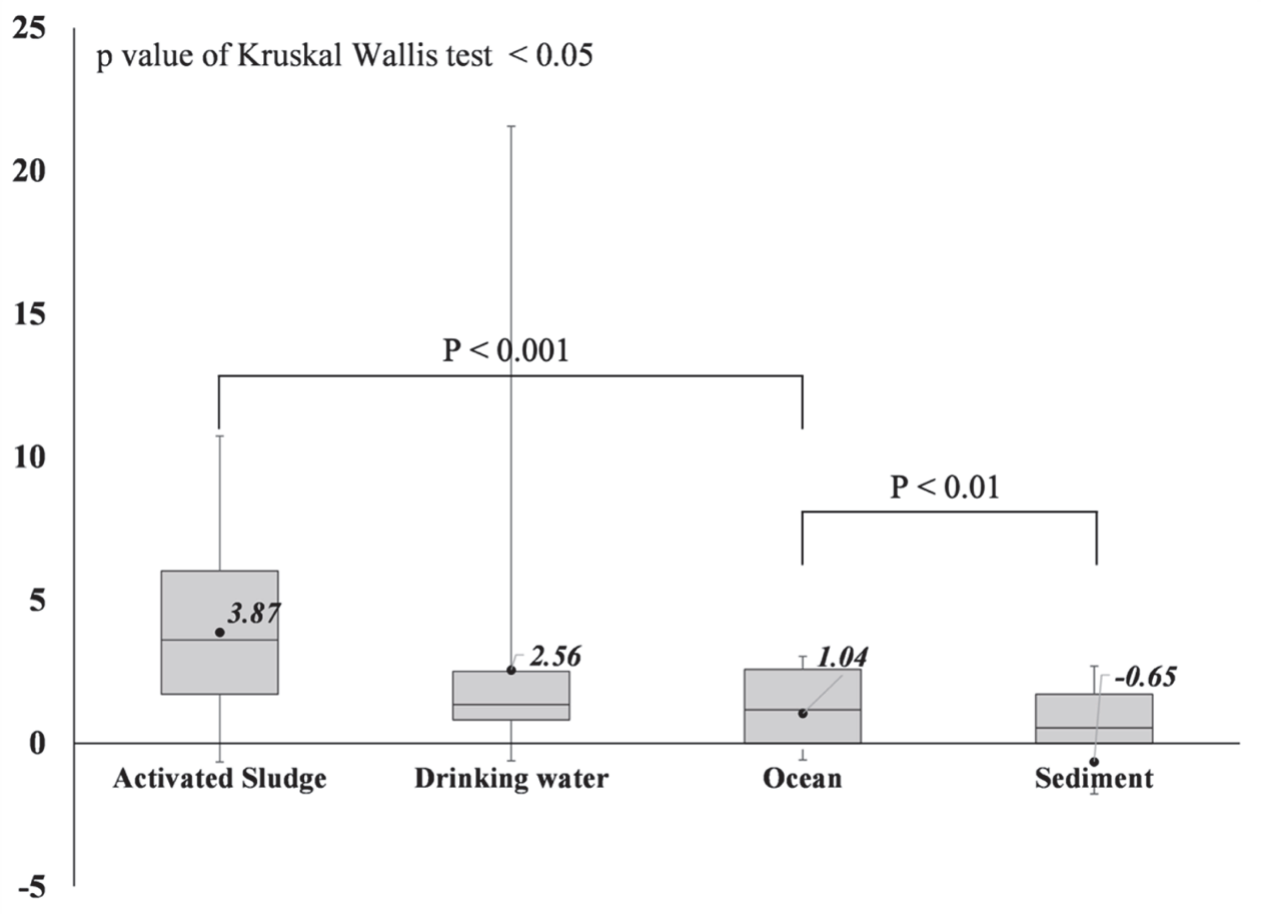
Estimated MPII values of 70 environmental metagenomes which were originated from four different environments. The MPII values of environmental metagenomes were estimated using Eq. (2). The top of the box is the seventy-fifth percentile, and the bottom of the box is the twenty-fifth percentile. The top and bottom whiskers represent the maximum and the minimum value, respectively. The black circle represents the mean value of the MPII within the environment. The statistical significance was calculated by the Kruskal–Wallis H test and post hoc Dunn’s test.

The MPII values of 70 environmental metagenomes were calculated using the suggested approach and equation (Fig. 2). The highest average MPII value showed in the activated sludge metagenomes, followed by drinking water, ocean, and sediment. The MPII values of the different environments were statistically distinguishable (H = 19.08; *p* < 0.001 by Kruskal-Wallis test). Dunn’s test showed that there was a significant difference between activated sludge metagenomes, which had the highest MPII values and ocean metagenomes (*p* < 0.001). In addition, ocean metagenomes and sediment metagenomes were also statistically distinguishable (*p* < 0.01). Overall, the average MPII value of activated sludge metagenomes was estimated to be 3.87, with a range of -0.65 to 10.73. The higher MPII values of the activated sludge metagenomes were mainly due to the detection of *Clostridium perfringens* and *Campylobacter jejuni* in Groups *VP*, *V*, and *P* (Fig. S4). The drinking water metagenomes had lower MPII values than activated sludge metagenomes (*i.e*., average 2.56). The MPII values of the drinking water metagenomes were associated with the detection of *Pseudomonas aeruginosa* and *Mycobacterium* genus, which were found in Groups *MVP* and *VP*. Among the environmental metagenomes, the lowest MPII values were found in the sediment metagenomes, with an average of -0.65.

The identification of pathogens in environmental metagenomes using our approach showed that each pathogen annotation group in our cumulative database had distinctive pathogens that affected the estimated MPII values. This result indicates that the suggested approach could be used not only to calculate the MPII values of metagenomes but also to identify the types of pathogens within a sample. The *Pseudomonas* genus (*P. mendocina,* *P. stutzeri, P. aeruginosa*), *Mycobacterium* genus (*M. smegmatis, M. tuberculosis, M. gilvum*), and *Clostridium botulinum* are highly associated with Group *MVP*. In Group *VP*, the *Chlamydia* genus, *Corynebacterium* genus, and *Bordetella* genus are present, together with many *Mycobacterium* species. The *Aeromonas* genus and *Rickettsia* genus are associated with Group *V*. Many species of the *Acinetobacter* genus and *Bacillus* genus were assigned in Groups *MP* and *P* (Fig. S4).

## Discussion

Our suggested approach for identifying pathogens in environmental metagenomes significantly improves the identification accuracy and the discriminating capacity compared to alignment to currently available databases. In addition, we provide an equation to calculate MPII, which is an intuitive index and enables sample-to-sample comparison of environmental metagenomes. The applicability of the suggested method was also demonstrated using 70 environmental metagenomes.

Through the evaluation of pathogen detection and identification using artificial metagenomes, the limitations of using a single database have been confirmed. The pathogen identification results using a single database showed lower sensitivity in its ability to discern pathogenic sequences from nonpathogenic sequences. For example, the nonpathogenic sequences originating or phylogenetically close from *Bacillus* were classified as pathogens by the VFDB and PATRIC database. In the studied metagenomes, the MLST database and the VFDB only could annotate up to 0.15% and 0.42% of total metagenomic sequences, respectively. In contrast, the PATRIC database was capable of annotating as many sequences as our suggested approach combining all three databases (Table 3). However, the sensitivity of the PATRIC database was not the highest although it requires the most computational requirements for annotation due to its large number of sequences.

The method proposed in this study differs from a single database-based pathogen identification method not only because this approach is capable of using as many sequences as possible but also because we did not consider equal weighting for pathogen-associated databases. For example, virulence factors are correlated with the expression of pathogenicity [58] and maybe a stronger indication of pathogenicity than an associated housekeeping gene. Thus, in the suggested approach, we utilized both the genetic information of a pathogen and the presence of specific virulence factors.

Group *MV*, which contains sequences detected by both the VFDB and the MLST database, was excluded from deriving an equation to calculate MPII because no sequence belonging to the group was identified in our artificial metagenomes. This result indicates that the sequences that can be detected by both the VFDB and MLST database can also be detected by the PATRIC database (*i.e*., Group *MVP*). In other words, if a sequence has an association with both housekeeping genes and virulence genes in the MLST database and VFDB, it is likely a well- documented pathogen and can be readily found in PATRIC database.

Several environmental metagenomes had lower MPII values than that of the artificial metagenome containing only nonpathogenic sequences (0.64). This result is associated with the observation that some nonpathogens are phylogenetically similar to the known pathogens and were intentionally included in the artificial metagenomes. Further, the microbial diversity of an environmental sample is much higher than that of the artificial metagenomes and results in a lower estimation of pathogenicity estimation.

In this study, an improved approach for metagenomic pathogen identification was provided by utilizing the currently available pathogen sequence databases more effectively. It was also confirmed that the abundance of pathogen sequences correlated with MPII values of tested metagenomes. The MPII equation derived using 100 pathogen and nonpathogen genomes showed statistical differences between environmental metagenomes originated from different environments. Among the four different environments, the metagenomes from activated sludge showed the highest MPII value on average. Importantly, the MPII values can be used for sample- to-sample comparison to get a brief but comprehensive understanding of how many the verified or suspicious pathogenic sequences existing in metagenomes, but it does not directly indicate the magnitude of the risk. Rather, the methodology suggested in this study was focused more on minimizing false negatives, enhancing the accuracy of identification, and providing an index that can be compared across metagenomes. As an early screening tool, the suggested approach can contribute to improving the ability to identify pathogens in the environment and complementing culture-based screening of indicator pathogens and existing molecular biological tools.

## Supplemental Materials



Supplementary data for this paper are available on-line only at http://jmb.or.kr.

## Figures and Tables

**Table 1:** Comparison of pathogen identification performance for artificial metagenomes between single database and suggested combination approach.

	MLST^a^	VFDB^b^	PATRIC^c^	Combination^d^
Sensitivity	0.74±0.00	0.91±0.01	0.82±0.00	0.96±0.00
Specificity	1.00±0.00	0.97±0.01	0.99±0.01	0.98±0.00
Prediction accuracy (AUC)	0.87±0.00	0.95±0.00	0.90±0.00	0.97±0.00

^a^MLST: annotated with only the MLST database, ^b^VFDB: with only the VFDB, ^c^PATRIC: with only the PATRIC database, ^d^Combination: with all the three databases

**Table 2:** Correlation coefficient and statistical significance of categorized groups determined by multiple linear regression analysis.

	MVP	VP	MV	V	MP	P	M
Correlation Coefficient	821.95	1.92	-	12.14	-63.20	1.37	292.66
*p*-value	< 0.0001	0.012	0.678	< 0.0001	< 0.0001	< 0.0001	< 0.0001
Intercept				-2.206			
Adjusted R^2^				0.884			

**Table 3:** Comparison of sequence usage for pathogen detection in 70 environmental metagenomes by the combination approach and each database. (Unit: %)

	MLST^a^	VFDB^b^	PATRIC^c^	Combination^d^
Average usage	0.08±0.03	0.07±0.02	3.95±2.76	3.97±2.77
Minimum usage	0.02	0.01	0.53	0.54
Maximum usage	0.15	0.42	19.26	19.31

^a^MLST: annotated with only the MLST database, ^b^VFDB: with only the VFDB, ^c^PATRIC: with only the PATRIC database, ^d^Combination: with all the three databases
