# Supplementary material for: An Improved Approach to Identify Bacterial Pathogens to Human in Environmental Metagenome
Source: J Microbiol Biotechnol. 2020 Jun 18;30(9):1335–42. doi: 10.4014/jmb.2005.05033 (PMC9728302; doi:10.4014/jmb.2005.05033)
Supplement: Supplementary file 1 [file JMB-30-9-1335-supple.pdf]

J. Microbiol. Biotechnol.
https://doi.org/10.4014/jmb.2005.05033

Table S1. Information of pathogens and non-pathogens included in artificial metagenomes.

Pathogen (n=50)	
Accession number	Name of bacteria
NC_000907.1	*Haemophilus influenzae* Rd KW20
NC_000915.1	*Helicobacter pylori* 26695
NC_000962.3	*Mycobacterium tuberculosis* H37Rv
NC_000963.1	*Rickettsia prowazekii* str. Madrid E
NC_002163.1	*Campylobacter jejuni* subsp. jejuni NCTC 11168 = ATCC 700819
NC_002505.1	*Vibrio cholerae* O1 biovar El Tor str. N16961
NC_002516.2	*Pseudomonas aeruginosa* PAO1
NC_002695.2	*Escherichia coli* O157:H7 str. Sakai DNA
NC_002737.2	*Streptococcus pyogenes* M1 GAS
NC_002929.2	*Bordetella pertussis* Tohama I
NC_002942.5	*Legionella pneumophila* subsp. pneumophila str. Philadelphia 1
NC_002971.4	*Coxiella burnetii* RSA 493
NC_003112.2	*Neisseria meningitidis* MC58
NC_003143.1	*Yersinia pestis* CO92
NC_003197.2	*Salmonella enterica* subsp. enterica serovar Typhimurium str. LT2
NC_003198.1	*Salmonella enterica* subsp. enterica serovar Typhi str. CT18
NC_003210.1	*Listeria monocytogenes* EGD-e
NC_003317.1	*Brucella melitensis* bv. 1 str. 16M
NC_003997.3	*Bacillus anthracis* str. Ames
NC_004116.1	*Streptococcus agalactiae* 2603V/R
NC_004310.3	*Brucella suis* 1330
NC_004337.2	*Shigella flexneri* 2a str. 301
NC_004459.3	*Vibrio vulnificus* CMCP6
NC_004461.1	*Staphylococcus epidermidis* ATCC 12228
NC_004722.1	*Bacillus cereus* ATCC 14579
NC_005956.1	*Bartonella henselae* strain Houston-1
NC_006155.1	*Yersinia pseudotuberculosis* IP32953
NC_006348.1	*Burkholderia mallei* ATCC 23344
NC_006350.1	*Burkholderia pseudomallei* K96243
NC_006570.2	*Francisella tularensis* subsp. tularensis SCHU S4
NC_006932.1	*Brucella abortus* biovar 1 str. 9-941
NC_007168.1	*Staphylococcus haemolyticus* JCSC1435
NC_007606.1	*Shigella dysenteriae* Sd197
NC_007795.1	*Staphylococcus aureus* subsp. aureus NCTC 8325
NC_007797.1	*Anaplasma phagocytophilum* HZ
NC_008261.1	*Clostridium perfringens* ATCC 13124
NC_008570.1	*Aeromonas hydrophila* subsp. hydrophila ATCC 7966
NC_008599.1	*Campylobacter fetus* subsp. fetus 82-40
NC_008800.1	*Yersinia enterocolitica* subsp. enterocolitica 8081
NC_009085.1	*Acinetobacter baumannii* ATCC 17978

NC_009089.1	*Clostridioides difficile* 630
NC_009698.1	*Clostridium botulinum* A str. Hall
NC_009715.2	*Campylobacter curvus* 525.92
NC_010103.1	*Brucella canis* ATCC 23365
NC_010287.1	*Chlamydia trachomatis* 434/Bu
NC_010503.1	*Ureaplasma parvum* serovar 3 str. ATCC 27815
NC_010673.1	*Borrelia hermsii* DAH
NC_011000.1	*Burkholderia cenocepacia* J2315
NC_011750.1	*Escherichia coli* IAI39
NC_015470.1	*Chlamydia psittaci* 6BC
Non-pathogen (n=50)	
Accession number	Name of bacteria
AP017991.1	*Planktothrix agardhii* NIES-204
CP003659.1	*Anabaena cylindrica* PCC 7122
CP013140.1	*Lysobacter enzymogenes* strain C3
CP035712.1	*Zymomonas mobilis* strain ZM4
LO018304.1	*Planktothrix agardhii* str. 7805
NC_001263.1	*Deinococcus radiodurans* R1
NC_002936.3	*Dehalococcoides mccartyi* 195
NC_002937.3	*Desulfovibrio vulgaris* str. Hildenborough
NC_003450.3	*Corynebacterium glutamicum* ATCC 13032
NC_004307.2	*Bifidobacterium longum* NCC2705
NC_004757.1	*Nitrosomonas europaea* ATCC 19718
NC_005877.1	*Picrophilus torridus* DSM 9790
NC_006055.1	*Mesoplasma florum* L1
NC_006814.3	*Lactobacillus acidophilus* NCFM
NC_007406.1	*Nitrobacter winogradskyi* Nb-255
NC_007413.1	*Anabaena variabilis* ATCC 29413
NC_007498.2	*Pelobacter carbinolicus* DSM 2380
NC_007575.1	*Sulfurimonas denitrificans* DSM 1251
NC_007576.1	*Lactobacillus sakei* strain 23K
NC_007761.1	*Rhizobium etli* CFN 42
NC_007963.1	*Chromohalobacter salexigens* DSM 3043
NC_008578.1	*Acidothermus cellulolyticus* 11B
NC_008609.1	*Pelobacter propionicus* DSM 2379
NC_008639.1	*Chlorobium phaeobacteroides* DSM 266
NC_008702.1	*Azoarcus* sp. BH72
NC_008786.1	*Verminephrobacter eiseniae* EF01-2
NC_009012.1	*Hungateiclostridium thermocellum* ATCC 27405
NC_009484.1	*Acidiphilium cryptum* JF-5
NC_009485.1	*Bradyrhizobium* sp. BTAi1
NC_009718.1	*Fervidobacterium nodosum* Rt17-B1
NC_009719.1	*Parvibaculum lavamentivorans* DS-1
NC_010163.1	*Acholeplasma laidlawii* PG-8A
NC_010296.1	*Microcystis aeruginosa* NIES-843

NC_010338.1	*Caulobacter* sp. K31
NC_010628.1	*Nostoc punctiforme* PCC 73102
NC_012491.1	*Brevibacillus brevis* NBRC 100599
NC_013410.1	*Fibrobacter succinogenes* subsp. succinogenes S85
NC_013552.1	*Dehalococcoides mccartyi* VS
NC_014219.1	*Bacillus selenitireducens* MLS10
NC_014638.1	*Bifidobacterium bifidum* PRL2010
NC_014666.1	*Frankia inefficax*
NC_015703.1	*Runella slithyformis* DSM 19594
NC_016943.1	*Blastococcus saxobsidens* DD2
NC_019673.1	Saccharothrix espanaensis DSM 44229
NC_019693.1	Oscillatoria acuminata PCC 6304
NZ_AP019314.1	Microcystis viridis NIES-102
NZ_CP007033.1	*Dehalobacter restrictus* DSM 9455
NZ_CP007445.1	*Gilliamella apicola* strain wkB1
NZ_CP011130.1	*Lysobacter capsici* strain 55
NZ_CP013187.1	*Pseudoalteromonas phenolica* strain KCTC 12086

Table S2. Information of public metagenome databases used in the present study.

Environment	Sample name	MG-RAST ID / NCBI-SRA ID	Number of reads	Total base pairs	Sequencing method
Wastewater activated sludge	W1	SRR2107210	25,162,108	3,774,316,200	Illumina/WGS
	W2	SRR2107211	28,187,818	4,228,172,700	Illumina/WGS
	W3	SRR2107218	31,034,732	4,655,209,800	Illumina/WGS
	W4	SRR2107219	29,177,086	4,376,562,900	Illumina/WGS
	W5	SRR2107215	30,330,608	4,549,591,200	Illumina/WGS
	W6	SRR2107216	29,950,702	4,492,605,300	Illumina/WGS
	W7	SRR2107212	17,799,468	2,669,920,200	Illumina/WGS
	W8	SRR2107213	24,813,164	3,721,974,600	Illumina/WGS
	W9	SRR2107184	32,511,436	4,876,715,400	Illumina/WGS
	W10	SRR2107185	17,290,848	2,593,627,200	Illumina/WGS
	W11	SRR2107182	30,824,764	4,623,714,600	Illumina/WGS
	W12	SRR2107183	31,628,652	4,744,297,800	Illumina/WGS
	W13	SRR2107180	20,992,566	3,148,884,900	Illumina/WGS
	W14	SRR2107181	29,012,192	4,351,828,800	Illumina/WGS
	W15	SRR2107178	20,128,328	3,019,249,200	Illumina/WGS
	W16	SRR2107179	21,681,186	3,252,177,900	Illumina/WGS
	W17	SRR2107176	25,354,668	3,803,200,200	Illumina/WGS
	W18	SRR2107177	28,772,996	4,315,949,400	Illumina/WGS
	W19	SRR2107171	24,924,044	3,738,606,600	Illumina/WGS
	W20	SRR2107173	29,967,076	4,495,061,400	Illumina/WGS
	W21	SRR2107174	25,262,572	3,789,385,800	Illumina/WGS
	W22	SRR2107175	23,844,798	3,576,719,700	Illumina/WGS
	W23	SRR2107128	31,935,134	4,790,270,100	Illumina/WGS
	W24	SRR2107133	28,282,146	4,242,321,900	Illumina/WGS
	W25	SRR609293	33,327,892	3,332,789,200	Illumina/WGS
	W26	SRR1702225	8,843,196	748,155,218	Illumina/WGS
	W27	SRR1702234	10,035,594	847,730,202	Illumina/WGS
	W28	SRR1702232	10,558,868	893,097,609	Illumina/WGS
	W29	SRR1702233	8,632,934	730,792,220	Illumina/WGS
	W30	SRR1106701	24,945,640	1,082,676,143	Illumina/WGS
	W31	SRR1106709	24,295,184	1,054,297,343	Illumina/WGS
	W32	SRR1106873	13,563,544	1,141,798,771	Illumina/WGS
Drinking water	D1	SRR6797127	39,836,630	3,983,663,000	Illumina/WGS
	D2	SRR6797128	36,922,136	3,692,213,600	Illumina/WGS
	D3	SRR6797129	40,637,918	4,063,791,800	Illumina/WGS
	D4	SRR6797130	47,747,352	4,774,735,200	Illumina/WGS
	D5	SRR6797131	34,692,986	3,469,298,600	Illumina/WGS
	D6	SRR6797132	39,104,158	3,910,415,800	Illumina/WGS
	D7	SRR6797133	37,492,672	3,749,267,200	Illumina/WGS
	D8	SRR6797134	39,982,660	3,998,266,000	Illumina/WGS
	D9	SRR6797135	39,122,576	3,912,257,600	Illumina/WGS
	D10	SRR6797136	40,956,934	4,095,693,400	Illumina/WGS
	D11	SRR6797137	39,014,288	3,901,428,800	Illumina/WGS
	D12	SRR6797138	40,228,356	4,022,835,600	Illumina/WGS
	D13	SRR6797139	38,981,940	3,898,194,000	Illumina/WGS
	D14	SRR6797140	39,345,326	3,934,532,600	Illumina/WGS
	D15	SRR6797141	42,892,530	4,289,253,000	Illumina/WGS
	D16	SRR6797142	46,173,050	4,617,305,000	Illumina/WGS
	D17	SRR6797143	39,313,886	3,931,388,600	Illumina/WGS
	D18	SRR6797144	37,523,972	3,752,397,200	Illumina/WGS
	D19	SRR6797145	42,526,778	4,252,677,800	Illumina/WGS
	D20	SRR6797146	34,484,024	3,448,402,400	Illumina/WGS
	D21	SRR6797147	40,273,488	4,027,348,800	Illumina/WGS
	D22	SRR6797148	43,754,266	4,375,426,600	Illumina/WGS
	D23	SRR6797149	39,931,246	3,993,124,600	Illumina/WGS
	D24	SRR6797150	45,790,870	4,579,087,000	Illumina/WGS
	D25	SRR6797151	40,878,198	4,087,819,800	Illumina/WGS
	D26	SRR850211	11,918,058	774,673,770	Illumina/WGS
	D27	SRR835363	11,907,016	773,956,040	Illumina/WGS
	D28	SRR850459	41,924,342	2,725,082,230	Illumina/WGS
	D29	SRR850212	34,555,046	2,246,077,990	Illumina/WGS
	S1	SRR1022373	27,962,564	2,796,256,400	Illumina/WGS
	S2	SRR1022377	27,381,826	2,738,182,600	Illumina/WGS

Sediment	S3	SRR1022378	29,889,520	2,988,952,000	Illumina/WGS
	S4	SRR1022348	29,761,346	2,976,134,600	Illumina/WGS
	S5	SRR1022349	26,561,056	2,656,105,600	Illumina/WGS
	O1	mgm4719971.3	1,655,635	388,842,875	Illumina/WGS
Ocean	O2	mgm4720028.3	1,293,725	366,286,427	Illumina/WGS
	O3	mgm4720058.3	1,426,897	602,118,955	Illumina/WGS
	O4	mgm4720034.3	2,406,292	271,339,155	Illumina/WGS

Table S3. The relative abundance of each categorized group in the 555 artificial metagenomes.

Sample name	Relative abundance (%)							
	MVP	VP	MV	V	MP	P	M	None
1_1	0.0019	0.0983	0.0000	0.0128	0.1295	5.0147	0.0114	94.7316
1_2	0.0012	0.0942	0.0000	0.0582	0.1260	4.6313	0.0101	95.0790
1_3	0.0012	0.1082	0.0001	0.0426	0.1300	4.6019	0.0120	95.1043
1_4	0.0013	0.0932	0.0000	0.1632	0.1257	4.5323	0.0113	95.0731
1_5	0.0015	0.0959	0.0000	0.0127	0.1290	5.1732	0.0111	94.5768
1_6	0.0012	0.1064	0.0000	0.0142	0.1258	5.4911	0.0102	94.2513
1_7	0.0011	0.1100	0.0000	0.0111	0.1264	5.0156	0.0100	94.7259
1_8	0.0012	0.0875	0.0000	0.0131	0.1262	4.9580	0.0116	94.8026
1_9	0.0013	0.1667	0.0000	0.0125	0.1391	5.6729	0.0120	93.9956
1_10	0.0014	0.1267	0.0000	0.0130	0.1265	5.4145	0.0108	94.3073
1_11	0.0014	0.1152	0.0000	0.0121	0.1305	5.2216	0.0105	94.5089
1_12	0.0011	0.1236	0.0000	0.0138	0.1260	5.3815	0.0107	94.3435
1_13	0.0012	0.1054	0.0000	0.0131	0.1321	4.9757	0.0107	94.7619
1_14	0.0019	0.2141	0.0000	0.0123	0.1263	5.8326	0.0113	93.8016
1_15	0.0012	0.1119	0.0000	0.0133	0.1255	5.4585	0.0105	94.2793
1_16	0.0014	0.1282	0.0000	0.0130	0.1234	4.8920	0.0122	94.8300
1_17	0.0012	0.1346	0.0000	0.0138	0.1295	5.1504	0.0118	94.5588
1_18	0.0011	0.1282	0.0000	0.0130	0.1251	4.9041	0.0100	94.8186
1_19	0.0010	0.1794	0.0000	0.0132	0.1283	5.5269	0.0105	94.1408
1_20	0.0016	0.1977	0.0000	0.0133	0.1251	5.4758	0.0108	94.1759
1_21	0.0009	0.1772	0.0000	0.0130	0.1366	5.5566	0.0106	94.1054
1_22	0.0021	0.1207	0.0000	0.0170	0.1303	4.9428	0.0096	94.7777
1_23	0.0009	0.1295	0.0001	0.0139	0.1249	5.1995	0.0104	94.5210
1_24	0.0012	0.1133	0.0000	0.0124	0.1282	5.7553	0.0104	93.9794
1_25	0.0007	0.1763	0.0000	0.0118	0.1315	5.4894	0.0103	94.1801
1_10_1	0.0015	0.1438	0.0000	0.0183	0.1288	5.1391	0.0106	94.5580
1_10_2	0.0013	0.1396	0.0000	0.0144	0.1275	5.3012	0.0098	94.4064
1_10_3	0.0011	0.1204	0.0000	0.0128	0.1286	5.2526	0.0107	94.4739
1_10_4	0.0012	0.1038	0.0000	0.0163	0.1287	4.8870	0.0103	94.8529
1_10_5	0.0015	0.1353	0.0000	0.0130	0.1225	5.1321	0.0124	94.5834
1_10_6	0.0015	0.1099	0.0000	0.0184	0.1281	5.1265	0.0107	94.6051
1_20_1	0.0010	0.1258	0.0000	0.0132	0.1270	5.2509	0.0106	94.4717
1_20_2	0.0013	0.1160	0.0000	0.0297	0.1301	5.0985	0.0096	94.6151
1_20_3	0.0013	0.1330	0.0000	0.0140	0.1240	5.1925	0.0121	94.5233
1_20_4	0.0010	0.1393	0.0000	0.0182	0.1300	5.2508	0.0111	94.4497
1_20_5	0.0016	0.1253	0.0000	0.0116	0.1291	5.2261	0.0115	94.4951
1_20_6	0.0012	0.1200	0.0000	0.0169	0.1346	5.0916	0.0106	94.6252
1_30_1	0.0013	0.1325	0.0001	0.0225	0.1243	5.1560	0.0108	94.5527
1_30_2	0.0011	0.1256	0.0000	0.0213	0.1219	5.1792	0.0114	94.5397
1_30_3	0.0012	0.1317	0.0001	0.0144	0.1250	5.1967	0.0110	94.5201
1_30_4	0.0013	0.1322	0.0000	0.0195	0.1245	5.1880	0.0109	94.5238
1_30_5	0.0013	0.1249	0.0000	0.0221	0.1299	5.0743	0.0116	94.6361
1_30_6	0.0010	0.1280	0.0000	0.0159	0.1362	5.1538	0.0107	94.5545
1_40_1	0.0016	0.1293	0.0000	0.0170	0.1305	5.1417	0.0111	94.5690
1_40_2	0.0012	0.1194	0.0001	0.0197	0.1255	5.1532	0.0104	94.5708
1_40_3	0.0016	0.1236	0.0000	0.0203	0.1269	5.1497	0.0100	94.5680
1_40_4	0.0011	0.1228	0.0000	0.0217	0.1302	5.0918	0.0108	94.6218

1_40_5	0.0010	0.1225	0.0000	0.0212	0.1282	5.1442	0.0103	94.5728
1_40_6	0.0015	0.1287	0.0000	0.0200	0.1277	5.1983	0.0108	94.5132
1_50_1	0.0011	0.1196	0.0000	0.0187	0.1270	5.1650	0.0112	94.5575
2_1	0.0013	0.1406	0.0000	0.0122	0.1265	5.8965	0.0110	93.8122
2_2	0.0017	0.2373	0.0001	0.0122	0.1333	6.4837	0.0098	93.1222
2_3	0.0015	0.1646	0.0001	0.0171	0.1257	5.3574	0.0112	94.3226
2_4	0.0009	0.1237	0.0000	0.0130	0.1290	6.0016	0.0104	93.7215
2_5	0.0013	0.1264	0.0001	0.0146	0.1291	5.4675	0.0119	94.2494
2_6	0.0014	0.0999	0.0000	0.1689	0.1282	4.9962	0.0104	94.5951
2_7	0.0016	0.1302	0.0001	0.0159	0.1312	5.1351	0.0105	94.5756
2_8	0.0013	0.1958	0.0000	0.0161	0.1331	5.9115	0.0109	93.7314
2_9	0.0011	0.1680	0.0000	0.0584	0.1254	5.0778	0.0108	94.5587
2_10	0.0018	0.2113	0.0000	0.0125	0.1310	6.6632	0.0104	92.9701
2_11	0.0012	0.1599	0.0000	0.0133	0.1234	5.6408	0.0109	94.0506
2_12	0.0011	0.1260	0.0000	0.0576	0.1353	5.5055	0.0111	94.1635
2_13	0.0009	0.1917	0.0000	0.0117	0.1343	6.2421	0.0102	93.4094
2_14	0.0009	0.1048	0.0000	0.0129	0.1277	5.6965	0.0120	94.0454
2_15	0.0009	0.1098	0.0001	0.0881	0.1318	4.6757	0.0124	94.9815
2_16	0.0012	0.1528	0.0000	0.0127	0.1245	5.8223	0.0114	93.8754
2_17	0.0016	0.1372	0.0000	0.0119	0.1229	5.4932	0.0104	94.2229
2_18	0.0010	0.1038	0.0000	0.0126	0.1279	5.3091	0.0104	94.4353
2_19	0.0017	0.2297	0.0000	0.0572	0.1352	5.6655	0.0108	93.9000
2_20	0.0016	0.1521	0.0000	0.0134	0.1308	6.2549	0.0106	93.4368
2_21	0.0011	0.1402	0.0000	0.0134	0.1339	5.3651	0.0107	94.3358
2_22	0.0012	0.1208	0.0000	0.0129	0.1291	5.5011	0.0112	94.2239
2_23	0.0014	0.1496	0.0000	0.0130	0.1328	6.1999	0.0112	93.4923
2_24	0.0015	0.2478	0.0000	0.0130	0.1324	6.1086	0.0118	93.4851
2_25	0.0012	0.1383	0.0000	0.0138	0.1255	5.5518	0.0097	94.1599
2_10_1	0.0021	0.1992	0.0000	0.0220	0.1291	5.7216	0.0124	93.9137
2_10_2	0.0011	0.1827	0.0000	0.0200	0.1325	6.0675	0.0119	93.5846
2_10_3	0.0013	0.1677	0.0000	0.0141	0.1347	6.0055	0.0117	93.6651
2_10_4	0.0013	0.1141	0.0000	0.0220	0.1269	5.2331	0.0107	94.4920
2_10_5	0.0010	0.1876	0.0000	0.0125	0.1285	5.7234	0.0123	93.9348
2_10_6	0.0013	0.1282	0.0000	0.0207	0.1313	5.6883	0.0116	94.0187
2_20_1	0.0011	0.1709	0.0000	0.0169	0.1286	5.9305	0.0124	93.7397
2_20_2	0.0012	0.1501	0.0000	0.0411	0.1302	5.6535	0.0119	94.0121
2_20_3	0.0015	0.1782	0.0000	0.0190	0.1324	5.7932	0.0110	93.8648
2_20_4	0.0019	0.1809	0.0001	0.0222	0.1292	5.9450	0.0108	93.7102
2_20_5	0.0013	0.1630	0.0000	0.0134	0.1286	5.9258	0.0105	93.7576
2_20_6	0.0016	0.1544	0.0000	0.0196	0.1327	5.6967	0.0117	93.9835
2_30_1	0.0013	0.1783	0.0000	0.0339	0.1314	5.7997	0.0112	93.8442
2_30_2	0.0014	0.1662	0.0000	0.0291	0.1291	5.8342	0.0118	93.8284
2_30_3	0.0015	0.1731	0.0001	0.0174	0.1292	5.8263	0.0116	93.8410
2_30_4	0.0011	0.1780	0.0000	0.0283	0.1277	5.8267	0.0108	93.8276
2_30_5	0.0013	0.1626	0.0000	0.0296	0.1304	5.5987	0.0102	94.0674
2_30_6	0.0014	0.1667	0.0000	0.0236	0.1348	5.7634	0.0105	93.8998
2_40_1	0.0013	0.1713	0.0000	0.0208	0.1301	5.7653	0.0108	93.9006
2_40_2	0.0014	0.1539	0.0000	0.0263	0.1338	5.7458	0.0125	93.9264
2_40_3	0.0010	0.1581	0.0000	0.0244	0.1278	5.7231	0.0111	93.9547
2_40_4	0.0014	0.1576	0.0000	0.0276	0.1333	5.6322	0.0112	94.0369
2_40_5	0.0015	0.1599	0.0000	0.0276	0.1299	5.7114	0.0093	93.9606

2_40_6	0.0012	0.1661	0.0000	0.0273	0.1287	5.7739	0.0113	93.8918
2_50_1	0.0012	0.1584	0.0000	0.0256	0.1301	5.7526	0.0118	93.9204
3_1	0.0011	0.1962	0.0000	0.0132	0.1331	6.7617	0.0119	92.8829
3_2	0.0012	0.1345	0.0000	0.0715	0.1280	5.7999	0.0122	93.8527
3_3	0.0018	0.1810	0.0000	0.0607	0.1293	5.5337	0.0137	94.0799
3_4	0.0015	0.1430	0.0000	0.0123	0.1318	7.4962	0.0094	92.2060
3_5	0.0015	0.1577	0.0000	0.0127	0.1257	6.2772	0.0111	93.4143
3_6	0.0015	0.1638	0.0000	0.0118	0.1300	5.8562	0.0103	93.8267
3_7	0.0015	0.1236	0.0000	0.1747	0.1242	5.3817	0.0102	94.1843
3_8	0.0013	0.1972	0.0000	0.0130	0.1405	7.2660	0.0108	92.3714
3_9	0.0011	0.1739	0.0000	0.0119	0.1297	6.0460	0.0112	93.6262
3_10	0.0009	0.2017	0.0000	0.0124	0.1301	6.1926	0.0112	93.4511
3_11	0.0013	0.2213	0.0000	0.0133	0.1332	6.3938	0.0110	93.2263
3_12	0.0013	0.1619	0.0000	0.0126	0.1318	7.1763	0.0108	92.5055
3_13	0.0019	0.2501	0.0001	0.0125	0.1251	6.7131	0.0106	92.8867
3_14	0.0015	0.1437	0.0000	0.0131	0.1286	6.2384	0.0106	93.4643
3_15	0.0012	0.2477	0.0000	0.0134	0.1365	6.9527	0.0105	92.6382
3_16	0.0016	0.1366	0.0001	0.0129	0.1347	5.6510	0.0117	94.0515
3_17	0.0013	0.1669	0.0000	0.0127	0.1275	5.8258	0.0100	93.8561
3_18	0.0012	0.2840	0.0000	0.0145	0.1401	6.9715	0.0108	92.5781
3_19	0.0013	0.1590	0.0000	0.0128	0.1334	6.3611	0.0115	93.3211
3_20	0.0013	0.2208	0.0000	0.0469	0.1289	6.3209	0.0116	93.2698
3_21	0.0009	0.1317	0.0001	0.1653	0.1287	5.7485	0.0104	93.8145
3_22	0.0015	0.1113	0.0000	0.0137	0.1290	6.1261	0.0121	93.6065
3_23	0.0015	0.1687	0.0000	0.0134	0.1283	6.2303	0.0092	93.4489
3_24	0.0012	0.1080	0.0000	0.0479	0.1269	5.6415	0.0116	94.0630
3_25	0.0016	0.2373	0.0000	0.0130	0.1328	6.5188	0.0090	93.0877
3_10_1	0.0014	0.2526	0.0000	0.0275	0.1299	6.3196	0.0120	93.2572
3_10_2	0.0014	0.2253	0.0000	0.0222	0.1341	6.7733	0.0119	92.8320
3_10_3	0.0011	0.2044	0.0001	0.0126	0.1341	6.7097	0.0102	92.9281
3_10_4	0.0011	0.1314	0.0000	0.0268	0.1339	5.5999	0.0120	94.0950
3_10_5	0.0019	0.2302	0.0000	0.0125	0.1350	6.3425	0.0100	93.2681
3_10_6	0.0011	0.1498	0.0000	0.0259	0.1280	6.2779	0.0105	93.4069
3_20_1	0.0012	0.2055	0.0001	0.0159	0.1333	6.6300	0.0109	93.0032
3_20_2	0.0016	0.1832	0.0000	0.0572	0.1316	6.2231	0.0119	93.3916
3_20_3	0.0012	0.2206	0.0000	0.0196	0.1329	6.4456	0.0109	93.1693
3_20_4	0.0013	0.2232	0.0000	0.0259	0.1328	6.6730	0.0112	92.9329
3_20_5	0.0013	0.1989	0.0001	0.0124	0.1315	6.6066	0.0111	93.0385
3_20_6	0.0011	0.1905	0.0000	0.0249	0.1312	6.2553	0.0114	93.3859
3_30_1	0.0015	0.2172	0.0000	0.0472	0.1293	6.3877	0.0108	93.2064
3_30_2	0.0011	0.2023	0.0000	0.0339	0.1347	6.5083	0.0117	93.1083
3_30_3	0.0015	0.2058	0.0000	0.0209	0.1292	6.5041	0.0114	93.1272
3_30_4	0.0012	0.2258	0.0000	0.0385	0.1369	6.4840	0.0102	93.1035
3_30_5	0.0010	0.1946	0.0000	0.0350	0.1283	6.1426	0.0115	93.4872
3_30_6	0.0014	0.2087	0.0000	0.0293	0.1336	6.3786	0.0107	93.2380
3_40_1	0.0009	0.2075	0.0000	0.0267	0.1375	6.3478	0.0117	93.2680
3_40_2	0.0017	0.1869	0.0000	0.0299	0.1298	6.3824	0.0108	93.2586
3_40_3	0.0016	0.1875	0.0000	0.0320	0.1314	6.3139	0.0115	93.3222
3_40_4	0.0012	0.1861	0.0000	0.0364	0.1341	6.2509	0.0118	93.3797
3_40_5	0.0010	0.1910	0.0000	0.0336	0.1313	6.3725	0.0105	93.2603
3_40_6	0.0010	0.1990	0.0000	0.0356	0.1354	6.4007	0.0115	93.2169

3_50_1	0.0016	0.1909	0.0000	0.0309	0.1351	6.3620	0.0108	93.2688
4_1	0.0012	0.2888	0.0000	0.0122	0.1434	7.7890	0.0097	91.7558
4_2	0.0010	0.1429	0.0000	0.0775	0.1273	6.3937	0.0119	93.2459
4_3	0.0018	0.1907	0.0000	0.0559	0.1294	5.9572	0.0127	93.6526
4_4	0.0014	0.2245	0.0000	0.0122	0.1408	8.6218	0.0108	90.9887
4_5	0.0010	0.1703	0.0000	0.0145	0.1319	6.7528	0.0113	92.9183
4_6	0.0013	0.1714	0.0000	0.0141	0.1284	6.7081	0.0115	92.9653
4_7	0.0013	0.1679	0.0001	0.1688	0.1304	6.0364	0.0120	93.4833
4_8	0.0016	0.2003	0.0000	0.0562	0.1378	7.3653	0.0101	92.2288
4_9	0.0010	0.1863	0.0000	0.0112	0.1287	6.5444	0.0105	93.1180
4_10	0.0014	0.2145	0.0000	0.0127	0.1296	6.6846	0.0101	92.9472
4_11	0.0016	0.2300	0.0000	0.0139	0.1385	6.9024	0.0106	92.7033
4_12	0.0013	0.1912	0.0000	0.0111	0.1393	7.8116	0.0111	91.8345
4_13	0.0010	0.2985	0.0000	0.0128	0.1274	7.0773	0.0114	92.4717
4_14	0.0015	0.2928	0.0000	0.0137	0.1402	7.1730	0.0110	92.3680
4_15	0.0016	0.3560	0.0000	0.0129	0.1476	7.9011	0.0111	91.5700
4_16	0.0012	0.1585	0.0000	0.0111	0.1348	6.8357	0.0118	92.8471
4_17	0.0007	0.2124	0.0001	0.0132	0.1334	6.4629	0.0120	93.1655
4_18	0.0021	0.2978	0.0000	0.0131	0.1366	7.4426	0.0112	92.0967
4_19	0.0012	0.2330	0.0000	0.0115	0.1356	7.3623	0.0103	92.2463
4_20	0.0016	0.2423	0.0000	0.0425	0.1367	7.5040	0.0115	92.0616
4_21	0.0011	0.1418	0.0000	0.1698	0.1332	6.6757	0.0115	92.8671
4_22	0.0019	0.1124	0.0000	0.0133	0.1315	6.9959	0.0126	92.7326
4_23	0.0015	0.1930	0.0000	0.0124	0.1295	6.7121	0.0111	92.9406
4_24	0.0017	0.1489	0.0000	0.0465	0.1296	6.2522	0.0115	93.4098
4_25	0.0011	0.2448	0.0000	0.0122	0.1349	6.5084	0.0104	93.0884
4_10_1	0.0015	0.3143	0.0000	0.0319	0.1377	6.8590	0.0114	92.6444
4_10_2	0.0014	0.2771	0.0001	0.0251	0.1377	7.5337	0.0109	92.0142
4_10_3	0.0018	0.2442	0.0000	0.0139	0.1370	7.4369	0.0104	92.1559
4_10_4	0.0012	0.1419	0.0000	0.0320	0.1284	5.9556	0.0105	93.7305
4_10_5	0.0017	0.2787	0.0000	0.0115	0.1320	6.9570	0.0108	92.6084
4_10_6	0.0012	0.1667	0.0000	0.0307	0.1338	6.8903	0.0112	92.7664
4_20_1	0.0015	0.2465	0.0000	0.0193	0.1383	7.3271	0.0112	92.2562
4_20_2	0.0013	0.2132	0.0000	0.0655	0.1367	6.7609	0.0105	92.8121
4_20_3	0.0017	0.2683	0.0001	0.0214	0.1362	7.0651	0.0109	92.4964
4_20_4	0.0012	0.2818	0.0000	0.0320	0.1403	7.3359	0.0103	92.1986
4_20_5	0.0016	0.2379	0.0000	0.0135	0.1348	7.3343	0.0112	92.2670
4_20_6	0.0018	0.2247	0.0000	0.0274	0.1328	6.8186	0.0114	92.7834
4_30_1	0.0014	0.2660	0.0000	0.0569	0.1371	7.0667	0.0117	92.4603
4_30_2	0.0019	0.2377	0.0001	0.0456	0.1353	7.1525	0.0115	92.4155
4_30_3	0.0012	0.2466	0.0000	0.0244	0.1339	7.1660	0.0103	92.4177
4_30_4	0.0017	0.2635	0.0000	0.0448	0.1345	7.1358	0.0114	92.4085
4_30_5	0.0015	0.2176	0.0000	0.0432	0.1309	6.6833	0.0111	92.9126
4_30_6	0.0015	0.2443	0.0000	0.0343	0.1357	7.0013	0.0110	92.5721
4_40_1	0.0014	0.2375	0.0000	0.0261	0.1357	6.9542	0.0118	92.6334
4_40_2	0.0011	0.2130	0.0000	0.0352	0.1363	6.9943	0.0108	92.6095
4_40_3	0.0015	0.2187	0.0000	0.0360	0.1349	6.9809	0.0108	92.6175
4_40_4	0.0012	0.2231	0.0000	0.0452	0.1365	6.8106	0.0109	92.7727
4_40_5	0.0014	0.2262	0.0000	0.0438	0.1336	6.9772	0.0111	92.6068
4_40_6	0.0013	0.2379	0.0001	0.0385	0.1398	7.0211	0.0123	92.5492
4_50_1	0.0015	0.2242	0.0000	0.0372	0.1351	6.9723	0.0106	92.6193

5_1	0.0015	0.3014	0.0000	0.0121	0.1385	8.3571	0.0112	91.1784
5_2	0.0016	0.1684	0.0000	0.0793	0.1314	7.0505	0.0128	92.5562
5_3	0.0014	0.2197	0.0000	0.0582	0.1327	6.3135	0.0127	93.2619
5_4	0.0014	0.3255	0.0000	0.0124	0.1409	9.5103	0.0110	89.9987
5_5	0.0013	0.1816	0.0000	0.0128	0.1328	6.9869	0.0098	92.6750
5_6	0.0014	0.1916	0.0000	0.0438	0.1312	6.7729	0.0103	92.8489
5_7	0.0014	0.1709	0.0000	0.1735	0.1365	6.0197	0.0113	93.4869
5_8	0.0019	0.2004	0.0000	0.0897	0.1372	7.3268	0.0120	92.2322
5_9	0.0017	0.1876	0.0000	0.0593	0.1352	6.6017	0.0100	93.0047
5_10	0.0011	0.2136	0.0000	0.0617	0.1302	6.7307	0.0121	92.8507
5_11	0.0014	0.2916	0.0001	0.0581	0.1427	6.9350	0.0115	92.5599
5_12	0.0016	0.2145	0.0000	0.0121	0.1372	8.3072	0.0112	91.3163
5_13	0.0012	0.3069	0.0000	0.0134	0.1296	7.3473	0.0096	92.1923
5_14	0.0016	0.2857	0.0000	0.1698	0.1370	7.1093	0.0116	92.2852
5_15	0.0013	0.3598	0.0000	0.0129	0.1382	8.7123	0.0103	90.7654
5_16	0.0017	0.1853	0.0000	0.0115	0.1377	7.5407	0.0098	92.1135
5_17	0.0017	0.3182	0.0000	0.0119	0.1299	7.3409	0.0105	92.1871
5_18	0.0013	0.3047	0.0000	0.0120	0.1336	8.0772	0.0103	91.4610
5_19	0.0009	0.2615	0.0000	0.0114	0.1391	8.5561	0.0111	91.0201
5_20	0.0017	0.2809	0.0000	0.0468	0.1378	7.8678	0.0116	91.6537
5_21	0.0012	0.1682	0.0001	0.1697	0.1340	7.3234	0.0103	92.1933
5_22	0.0018	0.2145	0.0000	0.0127	0.1276	7.8803	0.0120	91.7513
5_23	0.0017	0.2262	0.0000	0.0114	0.1311	7.4922	0.0111	92.1266
5_24	0.0013	0.1701	0.0000	0.0444	0.1294	7.4940	0.0114	92.1496
5_25	0.0016	0.2910	0.0000	0.0114	0.1358	7.5354	0.0104	92.0146
5_10_1	0.0015	0.3645	0.0000	0.0364	0.1313	7.4789	0.0125	91.9750
5_10_2	0.0014	0.3245	0.0000	0.0269	0.1446	8.2602	0.0107	91.2318
5_10_3	0.0011	0.2878	0.0000	0.0127	0.1369	8.2294	0.0116	91.3207
5_10_4	0.0012	0.1529	0.0000	0.0366	0.1347	6.2729	0.0131	93.3887
5_10_5	0.0019	0.3281	0.0000	0.0132	0.1345	7.5286	0.0110	91.9829
5_10_6	0.0014	0.1859	0.0000	0.0328	0.1337	7.4224	0.0099	92.2141
5_20_1	0.0015	0.2912	0.0000	0.0220	0.1398	8.0043	0.0111	91.5304
5_20_2	0.0013	0.2400	0.0000	0.0797	0.1342	7.3180	0.0119	92.2151
5_20_3	0.0018	0.3050	0.0000	0.0244	0.1355	7.7322	0.0104	91.7908
5_20_4	0.0013	0.3207	0.0000	0.0363	0.1398	8.0504	0.0102	91.4415
5_20_5	0.0009	0.2741	0.0000	0.0120	0.1362	8.0141	0.0111	91.5518
5_20_6	0.0015	0.2582	0.0000	0.0326	0.1410	7.4286	0.0117	92.1266
5_30_1	0.0014	0.3158	0.0000	0.0641	0.1351	7.6645	0.0096	91.8096
5_30_2	0.0018	0.2728	0.0001	0.0493	0.1340	7.8454	0.0118	91.6852
5_30_3	0.0012	0.2871	0.0000	0.0244	0.1349	7.8160	0.0111	91.7256
5_30_4	0.0013	0.3044	0.0001	0.0507	0.1418	7.7771	0.0115	91.7133
5_30_5	0.0011	0.2512	0.0001	0.0508	0.1319	7.2212	0.0128	92.3311
5_30_6	0.0013	0.2919	0.0000	0.0399	0.1380	7.6115	0.0115	91.9060
5_40_1	0.0012	0.2783	0.0000	0.0309	0.1361	7.5836	0.0111	91.9590
5_40_2	0.0009	0.2488	0.0000	0.0412	0.1371	7.5817	0.0103	91.9802
5_40_3	0.0012	0.2520	0.0000	0.0407	0.1314	7.5621	0.0107	92.0020
5_40_4	0.0016	0.2536	0.0000	0.0532	0.1400	7.3820	0.0119	92.1579
5_40_5	0.0015	0.2704	0.0000	0.0534	0.1374	7.5578	0.0099	91.9697
5_40_6	0.0014	0.2746	0.0000	0.0464	0.1352	7.6703	0.0112	91.8611
5_50_1	0.0015	0.2664	0.0000	0.0429	0.1347	7.5933	0.0116	91.9498
6_1	0.0010	0.3230	0.0000	0.0122	0.1469	9.5028	0.0093	90.0050

6_2	0.0007	0.1740	0.0000	0.0775	0.1342	7.0660	0.0117	92.5360
6_3	0.0019	0.2493	0.0000	0.0613	0.1362	7.0399	0.0113	92.5002
6_4	0.0012	0.3540	0.0000	0.0127	0.1430	10.2812	0.0089	89.1992
6_5	0.0013	0.2669	0.0000	0.0128	0.1442	8.0107	0.0099	91.5544
6_6	0.0020	0.1891	0.0000	0.0898	0.1324	6.8810	0.0114	92.6944
6_7	0.0013	0.1771	0.0000	0.1701	0.1354	6.2819	0.0111	93.2232
6_8	0.0014	0.3104	0.0001	0.0925	0.1416	8.2268	0.0130	91.2144
6_9	0.0016	0.1916	0.0000	0.0579	0.1370	7.4810	0.0115	92.1196
6_10	0.0015	0.2354	0.0000	0.0587	0.1352	7.6699	0.0116	91.8879
6_11	0.0012	0.3812	0.0000	0.0571	0.1465	7.9365	0.0113	91.4664
6_12	0.0015	0.2858	0.0000	0.0122	0.1462	9.4571	0.0115	90.0859
6_13	0.0010	0.3313	0.0001	0.0454	0.1302	7.4114	0.0114	92.0694
6_14	0.0023	0.3149	0.0000	0.1723	0.1366	7.4864	0.0106	91.8771
6_15	0.0014	0.3573	0.0000	0.1665	0.1449	8.6871	0.0105	90.6326
6_16	0.0015	0.2628	0.0000	0.0111	0.1431	8.6704	0.0104	90.9010
6_17	0.0016	0.3340	0.0000	0.0132	0.1338	7.8551	0.0111	91.6513
6_18	0.0014	0.3251	0.0000	0.0135	0.1417	8.7697	0.0105	90.7382
6_19	0.0015	0.3485	0.0000	0.0111	0.1422	9.5134	0.0098	89.9736
6_20	0.0014	0.3713	0.0000	0.0466	0.1405	8.8271	0.0111	90.6022
6_21	0.0013	0.1729	0.0000	0.1663	0.1328	7.6610	0.0117	91.8542
6_22	0.0012	0.2326	0.0000	0.0135	0.1310	8.4614	0.0118	91.1487
6_23	0.0014	0.2426	0.0000	0.0136	0.1309	8.0519	0.0105	91.5492
6_24	0.0014	0.1835	0.0000	0.0457	0.1329	7.9752	0.0110	91.6505
6_25	0.0022	0.3077	0.0000	0.0126	0.1321	8.0417	0.0111	91.4928
6_10_1	0.0018	0.4153	0.0000	0.0382	0.1389	8.0628	0.0124	91.3308
6_10_2	0.0011	0.3742	0.0000	0.0296	0.1492	9.0453	0.0106	90.3902
6_10_3	0.0011	0.3225	0.0000	0.0127	0.1444	8.9274	0.0119	90.5801
6_10_4	0.0009	0.1654	0.0000	0.0422	0.1282	6.6407	0.0113	93.0115
6_10_5	0.0011	0.3695	0.0001	0.0116	0.1355	8.1213	0.0096	91.3515
6_10_6	0.0014	0.2079	0.0000	0.0408	0.1359	8.0228	0.0113	91.5801
6_20_1	0.0010	0.3232	0.0000	0.0205	0.1383	8.6965	0.0095	90.8112
6_20_2	0.0015	0.2683	0.0000	0.0930	0.1349	7.8570	0.0102	91.6353
6_20_3	0.0013	0.3463	0.0000	0.0261	0.1387	8.4004	0.0103	91.0770
6_20_4	0.0014	0.3662	0.0001	0.0365	0.1395	8.7484	0.0107	90.6975
6_20_5	0.0018	0.3091	0.0001	0.0144	0.1406	8.6918	0.0112	90.8311
6_20_6	0.0018	0.2948	0.0000	0.0357	0.1385	8.0133	0.0106	91.5054
6_30_1	0.0014	0.3620	0.0000	0.0746	0.1372	8.2757	0.0110	91.1383
6_30_2	0.0016	0.3199	0.0000	0.0574	0.1420	8.5037	0.0122	90.9634
6_30_3	0.0017	0.3220	0.0000	0.0262	0.1382	8.4675	0.0103	91.0343
6_30_4	0.0013	0.3548	0.0001	0.0589	0.1416	8.4268	0.0107	91.0061
6_30_5	0.0015	0.2997	0.0001	0.0611	0.1356	7.7468	0.0107	91.7447
6_30_6	0.0014	0.3245	0.0000	0.0429	0.1381	8.2145	0.0094	91.2694
6_40_1	0.0014	0.3133	0.0000	0.0370	0.1435	8.1816	0.0111	91.3123
6_40_2	0.0012	0.2782	0.0000	0.0523	0.1387	8.2446	0.0108	91.2745
6_40_3	0.0009	0.2874	0.0000	0.0497	0.1399	8.2129	0.0107	91.2985
6_40_4	0.0014	0.2874	0.0000	0.0589	0.1388	7.9613	0.0117	91.5405
6_40_5	0.0013	0.3007	0.0000	0.0591	0.1402	8.1849	0.0116	91.3024
6_40_6	0.0015	0.3108	0.0000	0.0531	0.1396	8.3161	0.0108	91.1683
6_50_1	0.0012	0.3058	0.0000	0.0488	0.1343	8.1942	0.0102	91.3057
7_1	0.0012	0.3421	0.0000	0.0138	0.1476	10.4147	0.0087	89.0722
7_2	0.0013	0.1945	0.0000	0.0771	0.1354	7.7965	0.0119	91.7835

7_3	0.0017	0.3413	0.0000	0.0562	0.1425	8.0438	0.0121	91.4026
7_4	0.0017	0.3613	0.0001	0.1645	0.1389	10.2261	0.0108	89.0968
7_5	0.0012	0.3527	0.0000	0.0115	0.1416	8.9678	0.0104	90.5149
7_6	0.0018	0.2370	0.0000	0.0916	0.1366	7.5125	0.0125	92.0082
7_7	0.0013	0.1940	0.0001	0.1771	0.1355	6.5559	0.0106	92.9258
7_8	0.0013	0.3147	0.0000	0.2452	0.1355	8.1874	0.0120	91.1040
7_9	0.0014	0.2194	0.0000	0.0547	0.1338	7.8911	0.0111	91.6888
7_10	0.0016	0.2495	0.0000	0.0599	0.1369	7.9057	0.0104	91.6362
7_11	0.0014	0.4053	0.0001	0.0581	0.1420	8.5338	0.0120	90.8474
7_12	0.0011	0.3135	0.0000	0.0133	0.1463	10.2331	0.0098	89.2831
7_13	0.0018	0.3521	0.0000	0.0443	0.1306	8.3086	0.0108	91.1520
7_14	0.0016	0.3182	0.0000	0.1718	0.1375	7.8892	0.0109	91.4710
7_15	0.0019	0.4095	0.0000	0.1637	0.1396	9.1468	0.0091	90.1296
7_16	0.0018	0.2746	0.0000	0.0122	0.1466	9.2578	0.0101	90.2972
7_17	0.0015	0.3641	0.0001	0.0141	0.1339	8.6098	0.0118	90.8649
7_18	0.0014	0.3444	0.0000	0.0428	0.1436	8.8669	0.0129	90.5882
7_19	0.0014	0.3514	0.0000	0.0136	0.1405	9.7754	0.0099	89.7081
7_20	0.0017	0.3759	0.0000	0.0484	0.1375	9.3344	0.0112	90.0910
7_21	0.0014	0.1975	0.0000	0.2003	0.1365	7.7187	0.0118	91.7340
7_22	0.0017	0.2397	0.0000	0.0119	0.1348	8.9371	0.0118	90.6632
7_23	0.0015	0.2626	0.0000	0.0124	0.1359	9.3041	0.0098	90.2738
7_24	0.0011	0.1960	0.0000	0.0467	0.1418	8.5051	0.0112	91.0983
7_25	0.0018	0.3481	0.0000	0.0124	0.1415	8.3888	0.0104	91.0972
7_10_1	0.0016	0.4724	0.0000	0.0468	0.1389	8.6469	0.0111	90.6824
7_10_2	0.0016	0.4174	0.0000	0.0336	0.1506	9.7801	0.0096	89.6072
7_10_3	0.0015	0.3508	0.0000	0.0121	0.1476	9.6692	0.0098	89.8092
7_10_4	0.0012	0.1810	0.0000	0.0470	0.1363	7.0005	0.0132	92.6211
7_10_5	0.0014	0.4263	0.0000	0.0114	0.1398	8.7314	0.0105	90.6793
7_10_6	0.0017	0.2228	0.0001	0.0440	0.1416	8.6034	0.0100	90.9765
7_20_1	0.0013	0.3687	0.0000	0.0251	0.1381	9.4120	0.0118	90.0432
7_20_2	0.0014	0.3090	0.0000	0.1049	0.1364	8.4332	0.0105	91.0048
7_20_3	0.0014	0.3982	0.0000	0.0302	0.1415	8.9890	0.0095	90.4304
7_20_4	0.0020	0.4153	0.0000	0.0435	0.1481	9.5026	0.0112	89.8774
7_20_5	0.0012	0.3516	0.0000	0.0120	0.1497	9.3633	0.0124	90.1100
7_20_6	0.0015	0.3283	0.0000	0.0384	0.1484	8.5709	0.0120	90.9006
7_30_1	0.0016	0.3915	0.0000	0.0842	0.1416	8.8834	0.0113	90.4866
7_30_2	0.0015	0.3470	0.0001	0.0696	0.1454	9.1431	0.0112	90.2824
7_30_3	0.0014	0.3656	0.0000	0.0296	0.1423	9.1006	0.0108	90.3498
7_30_4	0.0016	0.3908	0.0001	0.0679	0.1406	9.0934	0.0099	90.2960
7_30_5	0.0015	0.3325	0.0000	0.0659	0.1406	8.2780	0.0112	91.1704
7_30_6	0.0016	0.3663	0.0000	0.0475	0.1403	8.8398	0.0121	90.5926
7_40_1	0.0014	0.3492	0.0000	0.0396	0.1382	8.8391	0.0108	90.6218
7_40_2	0.0018	0.3113	0.0000	0.0524	0.1419	8.8355	0.0117	90.6456
7_40_3	0.0014	0.3214	0.0000	0.0516	0.1417	8.8292	0.0120	90.6429
7_40_4	0.0010	0.3206	0.0000	0.0699	0.1383	8.5709	0.0115	90.8880
7_40_5	0.0016	0.3342	0.0000	0.0674	0.1377	8.7598	0.0106	90.6888
7_40_6	0.0015	0.3514	0.0000	0.0596	0.1418	8.9190	0.0108	90.5162
7_50_1	0.0014	0.3318	0.0000	0.0567	0.1405	8.7831	0.0115	90.6751
8_1	0.0014	0.4236	0.0000	0.0115	0.1549	11.4107	0.0101	87.9879
8_2	0.0014	0.3053	0.0001	0.0824	0.1374	8.6740	0.0143	90.7852
8_3	0.0016	0.3957	0.0000	0.0569	0.1431	9.0298	0.0132	90.3599

8_4	0.0019	0.4957	0.0000	0.1646	0.1485	11.0842	0.0114	88.0938
8_5	0.0018	0.4995	0.0000	0.0121	0.1540	9.8150	0.0108	89.5070
8_6	0.0019	0.2598	0.0000	0.0945	0.1418	7.8967	0.0121	91.5933
8_7	0.0017	0.2836	0.0001	0.1785	0.1419	7.5215	0.0110	91.8618
8_8	0.0015	0.3466	0.0001	0.2380	0.1373	8.6236	0.0126	90.6406
8_9	0.0019	0.2376	0.0000	0.0598	0.1366	8.3599	0.0102	91.1942
8_10	0.0017	0.2879	0.0000	0.0589	0.1359	8.7863	0.0121	90.7175
8_11	0.0014	0.4076	0.0000	0.2175	0.1452	8.5261	0.0111	90.6912
8_12	0.0015	0.3286	0.0000	0.0119	0.1483	10.7077	0.0103	88.7919
8_13	0.0012	0.3661	0.0000	0.0445	0.1361	9.0425	0.0103	90.3996
8_14	0.0017	0.3207	0.0000	0.2082	0.1370	7.9116	0.0114	91.4095
8_15	0.0012	0.4230	0.0000	0.1660	0.1441	9.4220	0.0098	89.8340
8_16	0.0015	0.3005	0.0001	0.0116	0.1485	10.0092	0.0119	89.5170
8_17	0.0014	0.4050	0.0000	0.0137	0.1432	9.2585	0.0106	90.1678
8_18	0.0014	0.3422	0.0000	0.0433	0.1450	9.2301	0.0118	90.2265
8_19	0.0011	0.3620	0.0000	0.0117	0.1447	10.6918	0.0102	88.7786
8_20	0.0011	0.4211	0.0000	0.0464	0.1390	9.7157	0.0115	89.6654
8_21	0.0010	0.2006	0.0001	0.2052	0.1369	8.5668	0.0112	90.8784
8_22	0.0016	0.2435	0.0000	0.0501	0.1340	8.9931	0.0121	90.5658
8_23	0.0013	0.2741	0.0000	0.0122	0.1405	9.7628	0.0099	89.7994
8_24	0.0024	0.2785	0.0000	0.0448	0.1487	9.6543	0.0110	89.8605
8_25	0.0020	0.3542	0.0000	0.0114	0.1402	8.7330	0.0116	90.7479
8_10_1	0.0016	0.5277	0.0000	0.0472	0.1426	9.2614	0.0104	90.0092
8_10_2	0.0012	0.4679	0.0000	0.0358	0.1517	10.5267	0.0113	88.8056
8_10_3	0.0014	0.3968	0.0000	0.0107	0.1457	10.3836	0.0110	89.0510
8_10_4	0.0014	0.1915	0.0000	0.0479	0.1375	7.3740	0.0129	92.2350
8_10_5	0.0009	0.4658	0.0000	0.0120	0.1450	9.3892	0.0109	89.9765
8_10_6	0.0015	0.2440	0.0000	0.0506	0.1428	9.1750	0.0107	90.3755
8_20_1	0.0015	0.4107	0.0000	0.0258	0.1474	10.1424	0.0114	89.2610
8_20_2	0.0011	0.3338	0.0000	0.1218	0.1467	8.9892	0.0110	90.3966
8_20_3	0.0013	0.4456	0.0000	0.0313	0.1469	9.6997	0.0105	89.6649
8_20_4	0.0018	0.4621	0.0000	0.0464	0.1431	10.1890	0.0119	89.1458
8_20_5	0.0018	0.3919	0.0001	0.0121	0.1499	10.0973	0.0116	89.3356
8_20_6	0.0017	0.3665	0.0001	0.0441	0.1438	9.1727	0.0122	90.2591
8_30_1	0.0013	0.4386	0.0000	0.0949	0.1444	9.5215	0.0125	89.7870
8_30_2	0.0015	0.3919	0.0000	0.0742	0.1402	9.8176	0.0135	89.5612
8_30_3	0.0016	0.3978	0.0000	0.0330	0.1428	9.7823	0.0120	89.6307
8_30_4	0.0020	0.4321	0.0000	0.0787	0.1455	9.7760	0.0114	89.5544
8_30_5	0.0015	0.3595	0.0000	0.0772	0.1411	8.9080	0.0111	90.5017
8_30_6	0.0012	0.4078	0.0000	0.0563	0.1475	9.4784	0.0117	89.8972
8_40_1	0.0016	0.3897	0.0000	0.0455	0.1419	9.4547	0.0125	89.9543
8_40_2	0.0014	0.3453	0.0000	0.0637	0.1435	9.4501	0.0120	89.9841
8_40_3	0.0012	0.3602	0.0000	0.0568	0.1346	9.4315	0.0107	90.0052
8_40_4	0.0008	0.3580	0.0000	0.0745	0.1391	9.1335	0.0115	90.2826
8_40_5	0.0017	0.3691	0.0000	0.0758	0.1419	9.3695	0.0114	90.0307
8_40_6	0.0009	0.3804	0.0000	0.0662	0.1430	9.5745	0.0116	89.8235
8_50_1	0.0014	0.3639	0.0000	0.0653	0.1411	9.3677	0.0110	90.0497
9_1	0.0013	0.4232	0.0000	0.0477	0.1536	11.4304	0.0099	87.9340
9_2	0.0013	0.3798	0.0001	0.0737	0.1450	9.8760	0.0127	89.5115
9_3	0.0016	0.4158	0.0001	0.0601	0.1473	9.3286	0.0117	90.0350
9_4	0.0020	0.5167	0.0000	0.1621	0.1474	11.6105	0.0100	87.5515

9_5	0.0014	0.5528	0.0000	0.0128	0.1592	10.8433	0.0095	88.4212
9_6	0.0016	0.2717	0.0000	0.0927	0.1415	8.1497	0.0112	91.3319
9_7	0.0013	0.3431	0.0000	0.1748	0.1441	8.5863	0.0100	90.7405
9_8	0.0017	0.3571	0.0000	0.2498	0.1405	8.6234	0.0127	90.6150
9_9	0.0024	0.2739	0.0000	0.0579	0.1439	9.2490	0.0097	90.2633
9_10	0.0014	0.3140	0.0000	0.0595	0.1417	9.4751	0.0106	89.9979
9_11	0.0017	0.4128	0.0000	0.2171	0.1432	9.2410	0.0106	89.9738
9_12	0.0011	0.3205	0.0000	0.0119	0.1480	10.7859	0.0112	88.7217
9_13	0.0016	0.4304	0.0000	0.0454	0.1315	9.6954	0.0113	89.6845
9_14	0.0017	0.3680	0.0001	0.2141	0.1391	8.5420	0.0111	90.7241
9_15	0.0015	0.4353	0.0000	0.1713	0.1441	10.0623	0.0099	89.1758
9_16	0.0012	0.2943	0.0000	0.0122	0.1460	10.4438	0.0099	89.0927
9_17	0.0019	0.4080	0.0000	0.0113	0.1414	9.6071	0.0093	89.8213
9_18	0.0016	0.3491	0.0000	0.0435	0.1412	9.5371	0.0110	89.9168
9_19	0.0013	0.4522	0.0000	0.0126	0.1537	11.6333	0.0103	87.7367
9_20	0.0016	0.4491	0.0000	0.0449	0.1343	10.4383	0.0129	88.9191
9_21	0.0015	0.2385	0.0000	0.1997	0.1392	8.9393	0.0116	90.4703
9_22	0.0014	0.2503	0.0001	0.0463	0.1319	9.6181	0.0124	89.9397
9_23	0.0014	0.2880	0.0000	0.0127	0.1458	10.2626	0.0102	89.2795
9_24	0.0018	0.3312	0.0000	0.0466	0.1473	10.0040	0.0116	89.4578
9_25	0.0016	0.4388	0.0000	0.0126	0.1480	9.7287	0.0111	89.6593
9_10_1	0.0018	0.5761	0.0000	0.0558	0.1424	9.8644	0.0110	89.3486
9_10_2	0.0015	0.5077	0.0000	0.0390	0.1562	11.2637	0.0102	88.0218
9_10_3	0.0017	0.4374	0.0000	0.0125	0.1471	11.1786	0.0104	88.2123
9_10_4	0.0018	0.1991	0.0000	0.0535	0.1361	7.7636	0.0130	91.8331
9_10_5	0.0020	0.5157	0.0000	0.0132	0.1467	9.9651	0.0104	89.3471
9_10_6	0.0014	0.2632	0.0001	0.0529	0.1468	9.7854	0.0104	89.7399
9_20_1	0.0017	0.4493	0.0000	0.0267	0.1451	10.8429	0.0121	88.5224
9_20_2	0.0013	0.3679	0.0000	0.1386	0.1434	9.5524	0.0121	89.7845
9_20_3	0.0012	0.4891	0.0000	0.0341	0.1441	10.3048	0.0111	89.0158
9_20_4	0.0013	0.5070	0.0000	0.0512	0.1521	10.8931	0.0121	88.3834
9_20_5	0.0021	0.4327	0.0000	0.0124	0.1542	10.8249	0.0116	88.5622
9_20_6	0.0013	0.3984	0.0000	0.0504	0.1506	9.7603	0.0113	89.6279
9_30_1	0.0014	0.4929	0.0000	0.1029	0.1513	10.1859	0.0125	89.0534
9_30_2	0.0016	0.4317	0.0000	0.0845	0.1499	10.4773	0.0102	88.8450
9_30_3	0.0016	0.4426	0.0000	0.0345	0.1463	10.4669	0.0101	88.8982
9_30_4	0.0013	0.4801	0.0000	0.0819	0.1493	10.4271	0.0110	88.8496
9_30_5	0.0014	0.4059	0.0000	0.0825	0.1401	9.4083	0.0119	89.9501
9_30_6	0.0017	0.4440	0.0000	0.0595	0.1507	10.1038	0.0121	89.2284
9_40_1	0.0012	0.4400	0.0000	0.0456	0.1507	10.0236	0.0109	89.3283
9_40_2	0.0013	0.3734	0.0000	0.0683	0.1489	10.1056	0.0111	89.2916
9_40_3	0.0013	0.3993	0.0000	0.0664	0.1416	10.0481	0.0111	89.3324
9_40_4	0.0012	0.3923	0.0001	0.0827	0.1457	9.7324	0.0109	89.6349
9_40_5	0.0016	0.3972	0.0000	0.0866	0.1466	9.9886	0.0108	89.3688
9_40_6	0.0020	0.4266	0.0000	0.0744	0.1442	10.2149	0.0119	89.1261
9_50_1	0.0016	0.4038	0.0000	0.0709	0.1404	10.0702	0.0128	89.3004
10_1	0.0016	0.4664	0.0000	0.0436	0.1578	12.1151	0.0093	87.2063
10_2	0.0012	0.3842	0.0000	0.0766	0.1390	10.5081	0.0119	88.8791
10_3	0.0019	0.4319	0.0000	0.0960	0.1502	9.3753	0.0118	89.9331
10_4	0.0015	0.5507	0.0000	0.1677	0.1457	11.9713	0.0103	87.1529
10_5	0.0016	0.5526	0.0000	0.0112	0.1625	11.1326	0.0110	88.1287

10_6	0.0017	0.2978	0.0000	0.0944	0.1461	9.0742	0.0123	90.3737
10_7	0.0014	0.3577	0.0000	0.1727	0.1461	9.0711	0.0114	90.2396
10_8	0.0012	0.3563	0.0000	0.2550	0.1359	9.1362	0.0123	90.1033
10_9	0.0022	0.2966	0.0000	0.0571	0.1437	10.1724	0.0112	89.3169
10_10	0.0014	0.4032	0.0000	0.0594	0.1457	10.5174	0.0112	88.8619
10_11	0.0012	0.4419	0.0001	0.2070	0.1486	10.1329	0.0123	89.0562
10_12	0.0015	0.4798	0.0000	0.0108	0.1566	11.6325	0.0094	87.7095
10_13	0.0015	0.4480	0.0000	0.0419	0.1379	10.5572	0.0118	88.8019
10_14	0.0015	0.4047	0.0001	0.2072	0.1436	8.9007	0.0113	90.3311
10_15	0.0020	0.5817	0.0000	0.1710	0.1525	10.8694	0.0112	88.2124
10_16	0.0020	0.3068	0.0000	0.0102	0.1454	11.2740	0.0094	88.2524
10_17	0.0011	0.4440	0.0000	0.0116	0.1395	10.4968	0.0102	88.8970
10_18	0.0016	0.3723	0.0000	0.0432	0.1431	9.9671	0.0109	89.4620
10_19	0.0010	0.4704	0.0000	0.0118	0.1528	12.1475	0.0095	87.2072
10_20	0.0012	0.4547	0.0000	0.0456	0.1422	10.9585	0.0111	88.3869
10_21	0.0015	0.2532	0.0000	0.1980	0.1382	9.4660	0.0113	89.9319
10_22	0.0016	0.3256	0.0000	0.0451	0.1436	10.7903	0.0131	88.6807
10_23	0.0015	0.3074	0.0000	0.0146	0.1485	10.6422	0.0106	88.8754
10_24	0.0019	0.3394	0.0000	0.0447	0.1442	10.9916	0.0122	88.4661
10_25	0.0014	0.4861	0.0000	0.0129	0.1422	10.1260	0.0103	89.2213
10_10_1	0.0017	0.6409	0.0000	0.0563	0.1496	10.4746	0.0106	88.6665
10_10_2	0.0018	0.5606	0.0001	0.0428	0.1575	12.0432	0.0107	87.1835
10_10_3	0.0018	0.4875	0.0000	0.0120	0.1551	11.8864	0.0110	87.4464
10_10_4	0.0015	0.2169	0.0000	0.0587	0.1347	8.1035	0.0123	91.4725
10_10_5	0.0014	0.5705	0.0001	0.0128	0.1428	10.6024	0.0109	88.6594
10_10_6	0.0015	0.2884	0.0001	0.0565	0.1493	10.4174	0.0111	89.0759
10_20_1	0.0016	0.4960	0.0000	0.0271	0.1500	11.5512	0.0122	87.7621
10_20_2	0.0016	0.3996	0.0000	0.1525	0.1441	10.1249	0.0123	89.1651
10_20_3	0.0012	0.5244	0.0000	0.0375	0.1482	11.0126	0.0107	88.2656
10_20_4	0.0018	0.5663	0.0000	0.0585	0.1536	11.5925	0.0117	87.6158
10_20_5	0.0015	0.4640	0.0000	0.0114	0.1560	11.5519	0.0121	87.8031
10_20_6	0.0010	0.4346	0.0000	0.0513	0.1424	10.3790	0.0122	88.9796
10_30_1	0.0013	0.5339	0.0000	0.1208	0.1538	10.8063	0.0114	88.3727
10_30_2	0.0014	0.4782	0.0000	0.0870	0.1508	11.1373	0.0111	88.1344
10_30_3	0.0019	0.4722	0.0000	0.0370	0.1472	11.1748	0.0104	88.1567
10_30_4	0.0013	0.5263	0.0000	0.0872	0.1464	11.0156	0.0112	88.2121
10_30_5	0.0020	0.4431	0.0000	0.0910	0.1459	9.9921	0.0112	89.3149
10_30_6	0.0016	0.4827	0.0000	0.0663	0.1531	10.7386	0.0110	88.5469
10_40_1	0.0016	0.4703	0.0000	0.0503	0.1519	10.6866	0.0110	88.6284
10_40_2	0.0012	0.4104	0.0000	0.0779	0.1470	10.7065	0.0122	88.6449
10_40_3	0.0016	0.4308	0.0000	0.0706	0.1512	10.6599	0.0122	88.6738
10_40_4	0.0010	0.4242	0.0000	0.0920	0.1415	10.3047	0.0111	89.0257
10_40_5	0.0016	0.4459	0.0001	0.0872	0.1434	10.6079	0.0099	88.7042
10_40_6	0.0014	0.4612	0.0000	0.0786	0.1475	10.8925	0.0131	88.4058
10_50_1	0.0016	0.4524	0.0000	0.0723	0.1471	10.6903	0.0109	88.6255
c_1	0.0012	0.1060	0.0000	0.0130	0.1301	5.5282	0.0133	94.2084
c_2	0.0014	0.1176	0.0000	0.0119	0.1303	6.3913	0.0107	93.3369
c_3	0.0011	0.1288	0.0000	0.0137	0.1299	7.3783	0.0104	92.3380
c_4	0.0007	0.1442	0.0000	0.0128	0.1303	8.2601	0.0111	91.4410
c_5	0.0010	0.1593	0.0000	0.0125	0.1336	9.2326	0.0116	90.4496
c_6	0.0008	0.1753	0.0001	0.0123	0.1394	10.1564	0.0108	89.5051

c_7	0.0014	0.1908	0.0000	0.0117	0.1395	11.0461	0.0089	88.6018
c_8	0.0013	0.1968	0.0000	0.0120	0.1375	11.9848	0.0102	87.6577
c_9	0.0009	0.2149	0.0000	0.0113	0.1345	12.9530	0.0105	86.6751
c_10	0.0008	0.2268	0.0000	0.0122	0.1367	13.8093	0.0096	85.8047
e_1	0.0014	0.1668	0.0000	0.0144	0.1293	5.7458	0.0110	93.9314
e_2	0.0010	0.2424	0.0000	0.0127	0.1378	6.8449	0.0108	92.7506
e_3	0.0008	0.3192	0.0000	0.0136	0.1451	8.0417	0.0111	91.4686
e_4	0.0011	0.3894	0.0000	0.0124	0.1521	9.1614	0.0105	90.2733
e_5	0.0016	0.4740	0.0000	0.0116	0.1634	10.2804	0.0112	89.0580
e_6	0.0015	0.5358	0.0000	0.0115	0.1663	11.4191	0.0103	87.8558
e_7	0.0012	0.6191	0.0000	0.0130	0.1677	12.5427	0.0111	86.6454
e_8	0.0018	0.6905	0.0000	0.0104	0.1816	13.7129	0.0096	85.3934
e_9	0.0016	0.7717	0.0000	0.0122	0.1866	14.7802	0.0097	84.2382
e_10	0.0023	0.8279	0.0000	0.0118	0.1903	15.9078	0.0085	83.0516
h_1	0.0015	0.1265	0.0000	0.0135	0.1271	4.8753	0.0109	94.8454
h_2	0.0012	0.1682	0.0000	0.0133	0.1246	5.2425	0.0103	94.4401
h_3	0.0017	0.2013	0.0000	0.0133	0.1283	5.5920	0.0093	94.0544
h_4	0.0019	0.2475	0.0000	0.0116	0.1276	5.9418	0.0104	93.6593
h_5	0.0019	0.2877	0.0000	0.0126	0.1320	6.3234	0.0111	93.2314
h_6	0.0022	0.3337	0.0000	0.0141	0.1288	6.6680	0.0113	92.8421
h_7	0.0028	0.3649	0.0000	0.0127	0.1305	7.0363	0.0111	92.4419
h_8	0.0029	0.4122	0.0000	0.0127	0.1298	7.4128	0.0107	92.0190
h_9	0.0033	0.4424	0.0000	0.0145	0.1335	7.7722	0.0110	91.6232
h_10	0.0032	0.4915	0.0000	0.0118	0.1304	8.1686	0.0102	91.1845
s_1	0.0009	0.1686	0.0000	0.0116	0.1346	5.5517	0.0115	94.1213
s_2	0.0014	0.2514	0.0000	0.0125	0.1373	6.5864	0.0098	93.0014
s_3	0.0011	0.3324	0.0000	0.0133	0.1467	7.5671	0.0113	91.9282
s_4	0.0014	0.4098	0.0000	0.0133	0.1531	8.5528	0.0108	90.8590
s_5	0.0016	0.4793	0.0000	0.0123	0.1548	9.5653	0.0088	89.7780
s_6	0.0012	0.5694	0.0000	0.0138	0.1548	10.5367	0.0103	88.7139
s_7	0.0014	0.6540	0.0000	0.0118	0.1678	11.5422	0.0102	87.6127
s_8	0.0015	0.7337	0.0000	0.0120	0.1748	12.5916	0.0109	86.4757
s_9	0.0019	0.7987	0.0000	0.0113	0.1766	13.5461	0.0101	85.4555
s_10	0.0014	0.8907	0.0000	0.0118	0.1827	14.4883	0.0088	84.4165
v_1	0.0007	0.1323	0.0000	0.0137	0.1293	5.1848	0.0099	94.5295
v_2	0.0013	0.1698	0.0000	0.0132	0.1313	5.8333	0.0111	93.8401
v_3	0.0013	0.2131	0.0000	0.0126	0.1366	6.4363	0.0095	93.1906
v_4	0.0012	0.2549	0.0000	0.0130	0.1416	7.1130	0.0114	92.4651
v_5	0.0015	0.3020	0.0000	0.0107	0.1481	7.7257	0.0099	91.8024
v_6	0.0011	0.3461	0.0000	0.0118	0.1515	8.3790	0.0116	91.0992
v_7	0.0012	0.3837	0.0000	0.0143	0.1521	9.0644	0.0098	90.3746
v_8	0.0012	0.4252	0.0001	0.0138	0.1607	9.6878	0.0094	89.7021
v_9	0.0012	0.4704	0.0000	0.0124	0.1642	10.3766	0.0101	88.9652
v_10	0.0010	0.5118	0.0000	0.0122	0.1646	11.0769	0.0106	88.2230
0	0.0014	0.0918	0.0000	0.0124	0.1271	4.5529	0.0108	95.2037
10	0.0016	0.4422	0.0000	0.0742	0.1487	10.6565	0.0111	88.6657
50	0.0029	2.0584	0.0000	0.3612	0.2478	38.6856	0.0119	58.6323
90	0.0045	4.1233	0.0000	0.7182	0.3816	73.9853	0.0121	20.7752
100	0.0058	4.6775	0.0000	0.8286	0.4092	84.2586	0.0115	9.8091

Table S4. Summary the results of MLR model performance analysis.

MLR performance evaluation	Subset	Statistical indices			
		R2	Adjusted R2	RMSE	Range of VIF
	Training	0.81	0.75	8.39	3.84-5.22
	Test	0.72	0.68	11.28	4.68-7.34

Figure S1. Phylogenetic placements of pathogenic and nonpathogenic bacteria in artificial metagenomes. The phylogenetic tree was constructed using the neighbor-joining method with 1,000 bootstrap replications based on the maximum composite likelihood model in MEGA X. (black circle(n=50): pathogen, clear circle(n=50): nonpathogen).

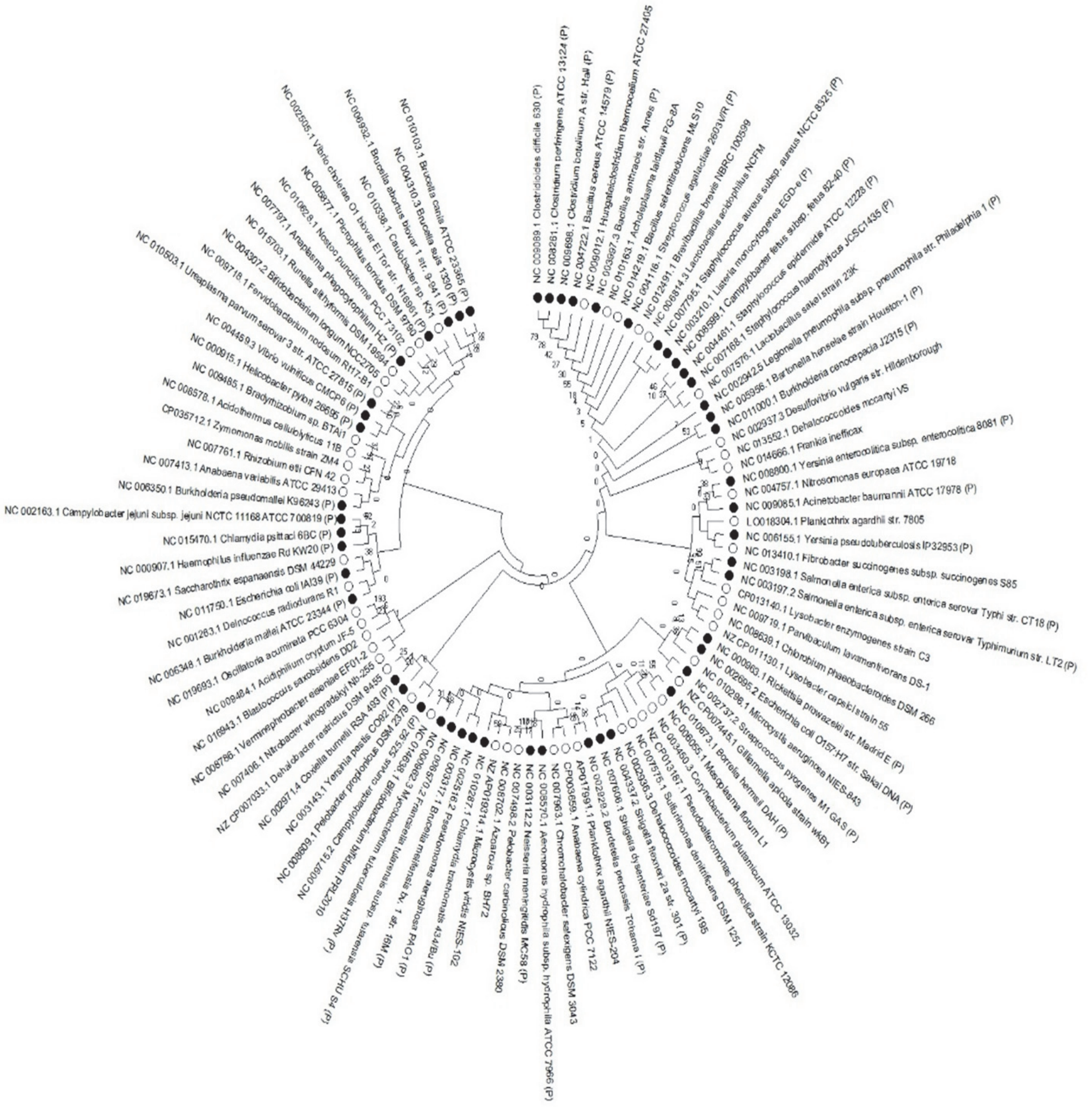

Figure S2. Categorization of metagenome sequences identified by the customized three databases. The categorization of metagenomic sequences is determined by which database identifies each query sequence. A sequence annotated by all three databases (the MLST, the VFDB, and the PATRIC) is included in Group MVP. Group MV contains sequences annotated by both the MLST database and the VFDB, and Group MP contains sequences annotated by both the MLST and the PATRIC database. In the same way, metagenomic sequences annotated by the VFDB and the PATRIC database are assigned to Group VP. When a sequence is annotated by a single database, it is included in Group M, V, and P. The sequences in the Group None indicates that any pathogen database could not identify those.

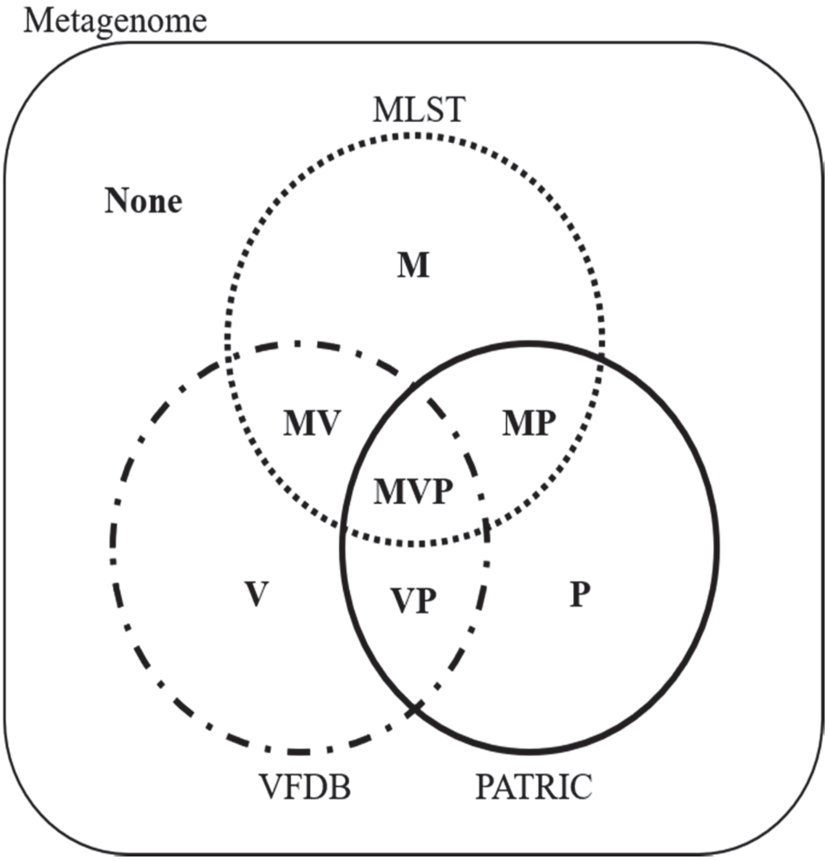

Figure S3. Pathogen identification results of 555 artificial metagenome. Pathogen identification was conducted using standalone BLASTN. VFDB, MLST, and PATRIC represented the utilized database, and the combination indicated that all three databases were used for the pathogen identification in the metagenome. The 0, 10, 50, 90, and 100 indicated the ratio of the pathogenic sequence in the artificial metagenome. The name of bacteria and circles at the right of a table represented the membership of artificial metagenome. The white circle indicated nonpathogens, and the black circle indicated pathogens. In the table, the white box indicated the bacteria marked on the right identified as nonpathogen by the database indicated above a table. The grey box meant the bacteria marked on the right of the table annotated as a pathogen.

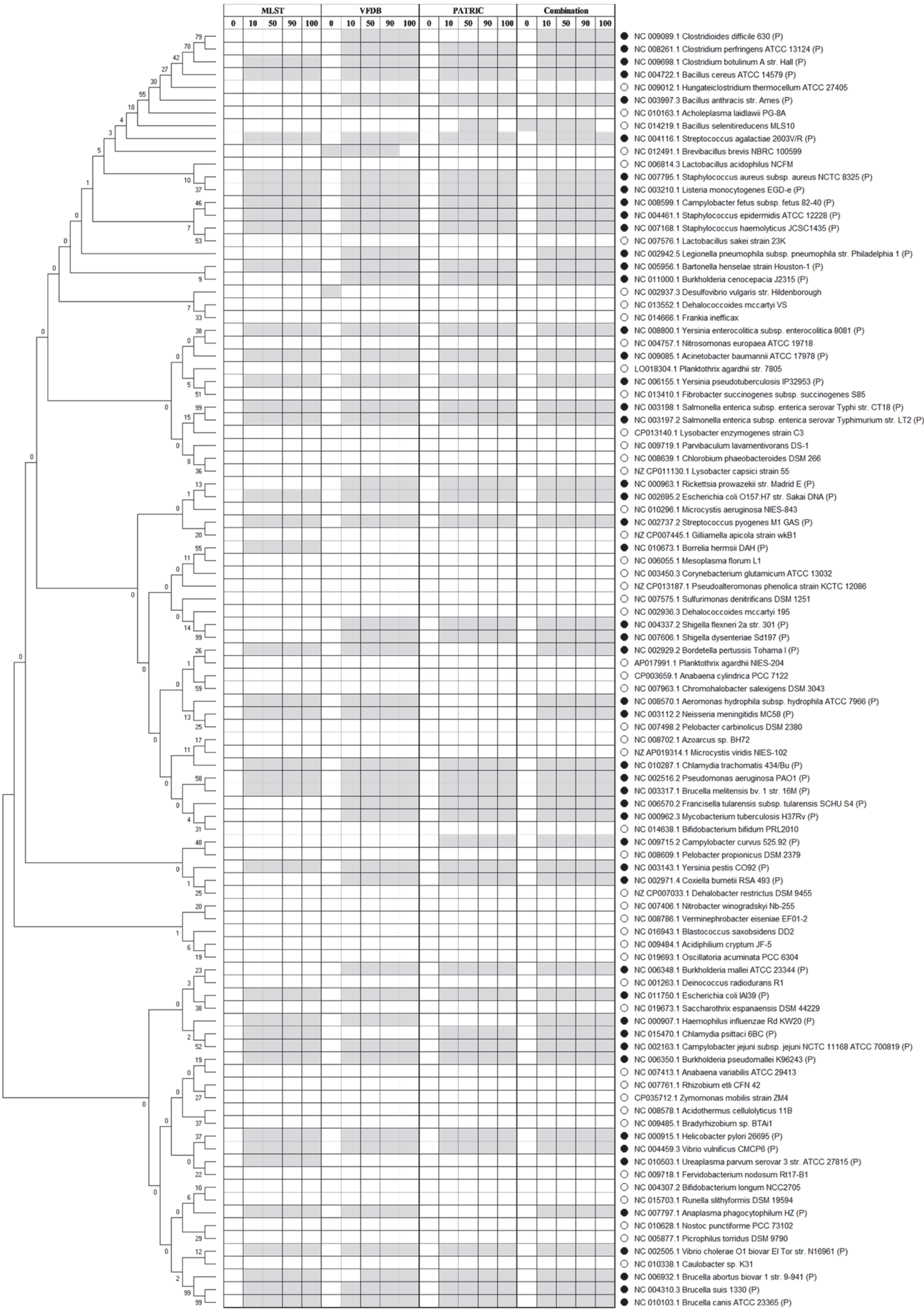

Figure S4. Identification of pathogens in environmental metagenomes. Pathogens in the environmental metagenomes were identified using standalone BLASTN, and the best match with the highest similarity was chosen for the following analysis. A sequence in the environmental metagenomes annotated by all three databases was included in Group MVP. The initials M, V, and P indicates that sequences were annotated by one of each MLST database, VFDB, and PATRIC database. The black boxes represent each bacterial species (or genus) identified in each group.

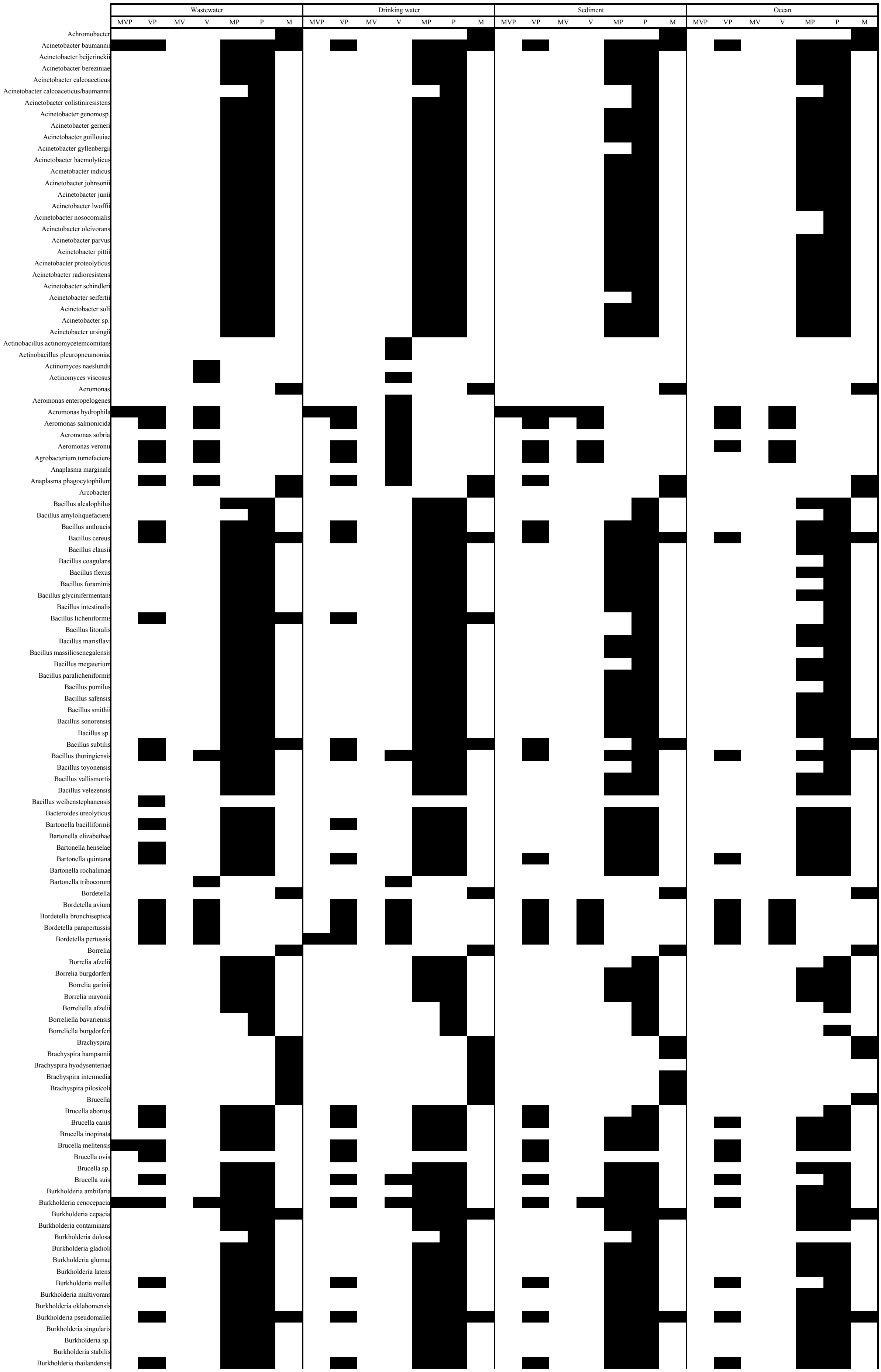

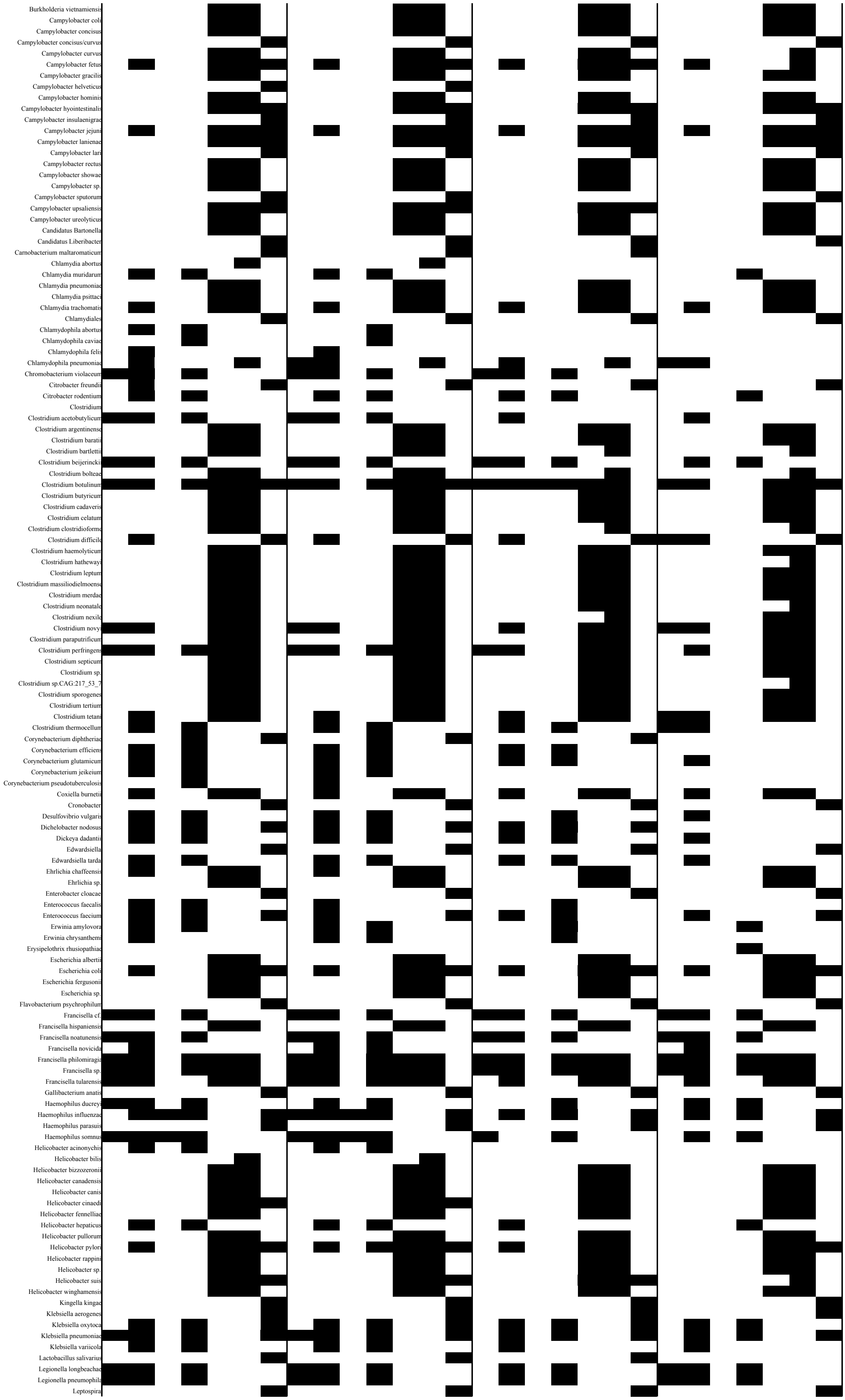
Burkholderia vietnamiensis
Campylobacter coli
Campylobacter concisus
Campylobacter concisus/curvus
Campylobacter curvus
Campylobacter fetus
Campylobacter gracilis
Campylobacter helveticus
Campylobacter hominis
Campylobacter hyointestinalis
Campylobacter insulaenigrae
Campylobacter jejuni
Campylobacter lanienae
Campylobacter lari
Campylobacter rectus
Campylobacter showae
Campylobacter sp.
Campylobacter sputorum
Campylobacter upsaliensis
Campylobacter ureolyticus
Candidatus Bartonella
Candidatus Liberibacter
Carnobacterium maltaromaticum
Chlamydia abortus
Chlamydia muridarum
Chlamydia pneumoniae
Chlamydia psittaci
Chlamydia trachomatis
Chlamydiales
Chlamydophila abortus
Chlamydophila caviae
Chlamydophila felis
Chlamydophila pneumoniae
Chromobacterium violaceum
Citrobacter freundii
Citrobacter rodentium
Clostridium
Clostridium acetobutylicum
Clostridium argentinense
Clostridium baratii
Clostridium bartlettii
Clostridium beijerinckii
Clostridium bolteae
Clostridium botulinum
Clostridium butyricum
Clostridium cadaveris
Clostridium celatum
Clostridium clostridioforme
Clostridium difficile
Clostridium haemolyticum
Clostridium hathewayi
Clostridium leptum
Clostridium massiliodielmoense
Clostridium merdae
Clostridium neonatale
Clostridium nexile
Clostridium novyi
Clostridium paraputrificum
Clostridium perfringens
Clostridium septicum
Clostridium sp.
Clostridium sp.CAG:217_53_7
Clostridium sporogenes
Clostridium tertium
Clostridium tetani
Clostridium thermocellum
Corynebacterium diphtheriae
Corynebacterium efficiens
Corynebacterium glutamicum
Corynebacterium jeikeium
Corynebacterium pseudotuberculosis
Coxiella burnetii
Cronobacter
Desulfovibrio vulgaris
Dichelobacter nodosus
Dickeya dadantii
Edwardsiella
Edwardsiella tarda
Ehrlichia chaffeensis
Ehrlichia sp.
Enterobacter cloacae
Enterococcus faecalis
Enterococcus faecium
Erwinia amylovora
Erwinia chrysanthemi
Erysipelothrix rhusiopathiae
Escherichia albertii
Escherichia coli
Escherichia fergusonii
Escherichia sp.
Flavobacterium psychrophilum
Francisella cf.
Francisella hispaniensis
Francisella noatunensis
Francisella novicida
Francisella philomiragia
Francisella sp.
Francisella tularensis
Gallibacterium anatis
Haemophilus ducreyi
Haemophilus influenzae
Haemophilus parasuis
Haemophilus somnus
Helicobacter acinonychis
Helicobacter bilis
Helicobacter bizzozeronii
Helicobacter canadensis
Helicobacter canis
Helicobacter cinaedi
Helicobacter fennelliae
Helicobacter hepaticus
Helicobacter pullorum
Helicobacter pylori
Helicobacter rappini
Helicobacter sp.
Helicobacter suis
Helicobacter winghamensis
Kingella kingae
Klebsiella aerogenes
Klebsiella oxytoca
Klebsiella pneumoniae
Klebsiella variicola
Lactobacillus salivarius
Legionella longbeachae
Legionella pneumophila
Leptospira

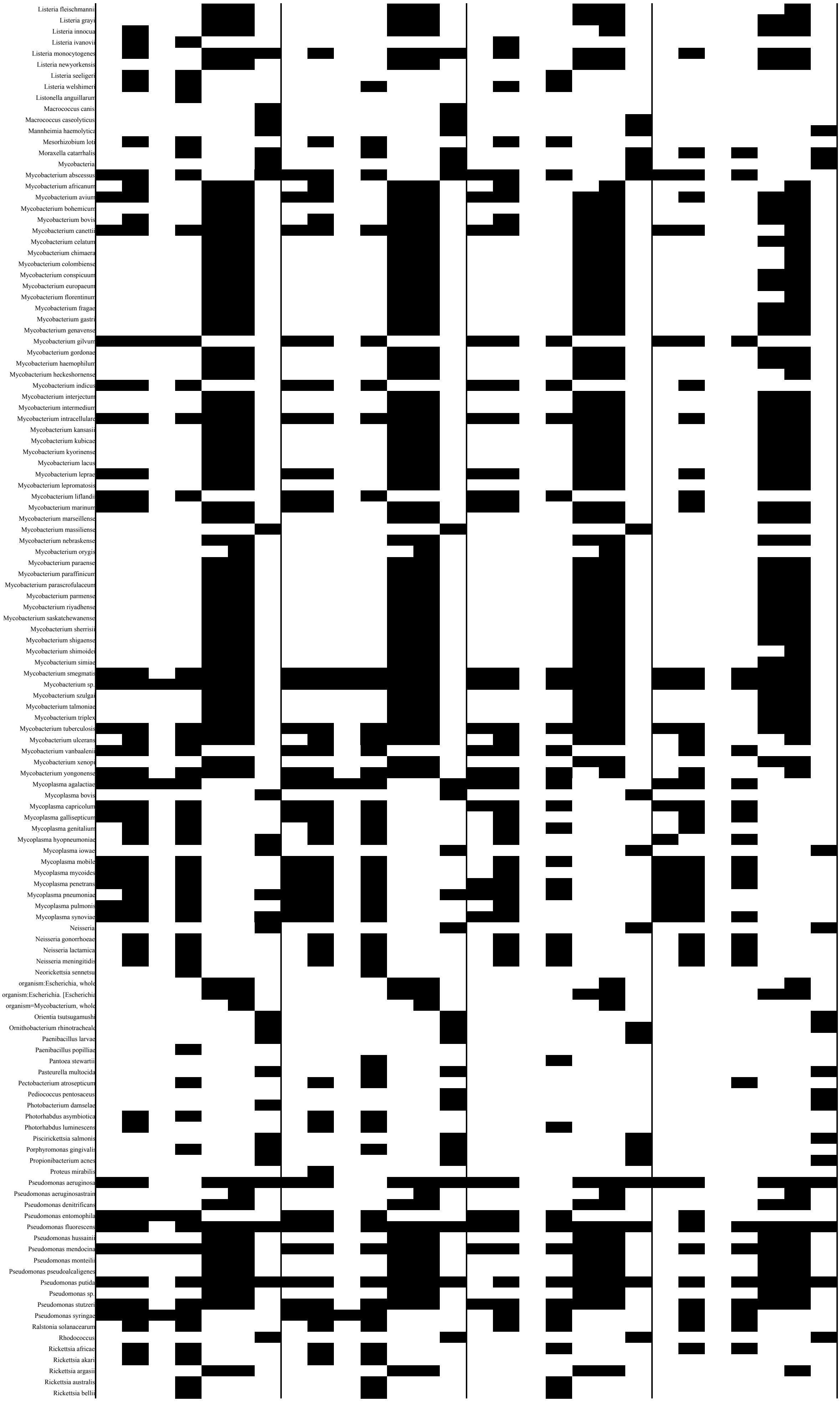

Listeria fleischmannii
Listeria grayi
Listeria innocua
Listeria ivanovii
Listeria monocytogenes
Listeria newyorkensis
Listeria seeligeri
Listeria welshimeri
Listonella anguillarum
Macrococcus canis
Macrococcus caseolyticus
Mannheimia haemolytica
Mesorhizobium loti
Moraxella catarrhalis
Mycobacteria
Mycobacterium abscessus
Mycobacterium africanum
Mycobacterium avium
Mycobacterium bohemicum
Mycobacterium bovis
Mycobacterium canettii
Mycobacterium celatum
Mycobacterium chimaera
Mycobacterium colombiense
Mycobacterium conspicuum
Mycobacterium europaeum
Mycobacterium florentinum
Mycobacterium fragae
Mycobacterium gastri
Mycobacterium genavense
Mycobacterium gilvum
Mycobacterium gordonae
Mycobacterium haemophilum
Mycobacterium heckeshornense
Mycobacterium indicus
Mycobacterium interjectum
Mycobacterium intermedium
Mycobacterium intracellulare
Mycobacterium kansasii
Mycobacterium kubicae
Mycobacterium kyorinense
Mycobacterium lacus
Mycobacterium leprae
Mycobacterium lepromatosis
Mycobacterium liflandii
Mycobacterium marinum
Mycobacterium marseillense
Mycobacterium massiliense
Mycobacterium nebraskense
Mycobacterium orygis
Mycobacterium paraense
Mycobacterium paraffinicum
Mycobacterium parascrofulaceum
Mycobacterium parmense
Mycobacterium riyadhense
Mycobacterium saskatchewanense
Mycobacterium sherrisii
Mycobacterium shigaense
Mycobacterium shimoidei
Mycobacterium simiae
Mycobacterium smegmatis
Mycobacterium sp.
Mycobacterium szulgai
Mycobacterium talmoniae
Mycobacterium triplex
Mycobacterium tuberculosis
Mycobacterium ulcerans
Mycobacterium vanbaalenii
Mycobacterium xenopi
Mycobacterium yongonense
Mycoplasma agalactiae
Mycoplasma bovis
Mycoplasma capricolum
Mycoplasma gallisepticum
Mycoplasma genitalium
Mycoplasma hyopneumoniae
Mycoplasma iowae
Mycoplasma mobile
Mycoplasma mycoides
Mycoplasma penetrans
Mycoplasma pneumoniae
Mycoplasma pulmonis
Mycoplasma synoviae
Neisseria
Neisseria gonorrhoeae
Neisseria lactamica
Neisseria meningitidis
Neorickettsia sennetsu
organism:Escherichia, whole
organism:Escherichia. [Escherichia
organism=Mycobacterium, whole
Orientia tsutsugamushi
Ornithobacterium rhinotracheale
Paenibacillus larvae
Paenibacillus popilliae
Pantoea stewartii
Pasteurella multocida
Pectobacterium atrosepticum
Pediococcus pentosaceus
Photobacterium damselae
Photorhabdus asymbiotica
Photorhabdus luminescens
Piscirickettsia salmonis
Porphyromonas gingivalis
Propionibacterium acnes
Proteus mirabilis
Pseudomonas aeruginosa
Pseudomonas aeruginosastrain
Pseudomonas denitrificans
Pseudomonas entomophila
Pseudomonas fluorescens
Pseudomonas hussainii
Pseudomonas mendocina
Pseudomonas monteilii
Pseudomonas pseudoalcaligenes
Pseudomonas putida
Pseudomonas sp.
Pseudomonas stutzeri
Pseudomonas syringae
Ralstonia solanacearum
Rhodococcus
Rickettsia africae
Rickettsia akari
Rickettsia argasii
Rickettsia australis
Rickettsia bellii

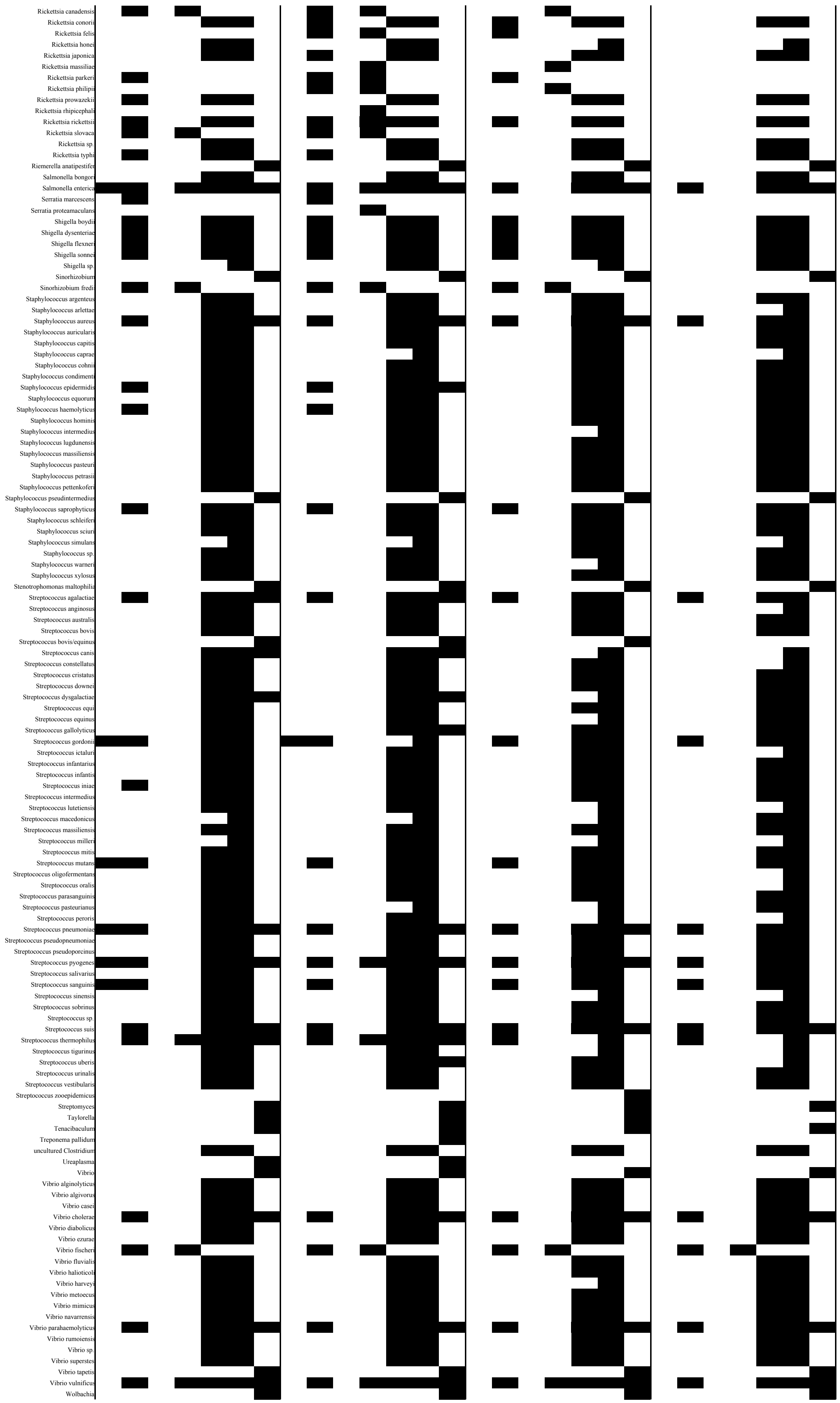
Rickettsia canadensis
Rickettsia conorii
Rickettsia felis
Rickettsia honei
Rickettsia japonica
Rickettsia massiliae
Rickettsia parkeri
Rickettsia philipii
Rickettsia prowazekii
Rickettsia rhipicephali
Rickettsia rickettsii
Rickettsia slovaca
Rickettsia sp.
Rickettsia typhi
Riemerella anatipestifer
Salmonella bongori
Salmonella enterica
Serratia marcescens
Serratia proteamaculans
Shigella boydii
Shigella dysenteriae
Shigella flexneri
Shigella sonnei
Shigella sp.
Sinorhizobium
Sinorhizobium fredii
Staphylococcus argenteus
Staphylococcus arlettae
Staphylococcus aureus
Staphylococcus auricularis
Staphylococcus capitis
Staphylococcus caprae
Staphylococcus cohnii
Staphylococcus condimenti
Staphylococcus epidermidis
Staphylococcus equorum
Staphylococcus haemolyticus
Staphylococcus hominis
Staphylococcus intermedius
Staphylococcus lugdunensis
Staphylococcus massiliensis
Staphylococcus pasteuri
Staphylococcus petrasii
Staphylococcus pettenkoferi
Staphylococcus pseudintermedius
Staphylococcus saprophyticus
Staphylococcus schleiferi
Staphylococcus sciuri
Staphylococcus simulans
Staphylococcus sp.
Staphylococcus warneri
Staphylococcus xylosus
Stenotrophomonas maltophilia
Streptococcus agalactiae
Streptococcus anginosus
Streptococcus australis
Streptococcus bovis
Streptococcus bovis/equinus
Streptococcus canis
Streptococcus constellatus
Streptococcus cristatus
Streptococcus downei
Streptococcus dysgalactiae
Streptococcus equi
Streptococcus equinus
Streptococcus gallolyticus
Streptococcus gordonii
Streptococcus ictaluri
Streptococcus infantarius
Streptococcus infantis
Streptococcus iniae
Streptococcus intermedius
Streptococcus lutetiensis
Streptococcus macedonicus
Streptococcus massiliensis
Streptococcus milleri
Streptococcus mitis
Streptococcus mutans
Streptococcus oligofermentans
Streptococcus oralis
Streptococcus parasanguinis
Streptococcus pasteurianus
Streptococcus peroris
Streptococcus pneumoniae
Streptococcus pseudopneumoniae
Streptococcus pseudoporcinus
Streptococcus pyogenes
Streptococcus salivarius
Streptococcus sanguinis
Streptococcus sinensis
Streptococcus sobrinus
Streptococcus sp.
Streptococcus suis
Streptococcus thermophilus
Streptococcus tigurinus
Streptococcus uberis
Streptococcus urinalis
Streptococcus vestibularis
Streptococcus zooepidemicus
Streptomyces
Taylorella
Tenacibaculum
Treponema pallidum
uncultured Clostridium
Ureaplasma
Vibrio
Vibrio alginolyticus
Vibrio algivorus
Vibrio casei
Vibrio cholerae
Vibrio diabolicus
Vibrio ezurae
Vibrio fischeri
Vibrio fluvialis
Vibrio halioticoli
Vibrio harveyi
Vibrio metoecus
Vibrio mimicus
Vibrio navarrensis
Vibrio parahaemolyticus
Vibrio rumoiensis
Vibrio sp.
Vibrio superstes
Vibrio tapetis
Vibrio vulnificus
Wolbachia

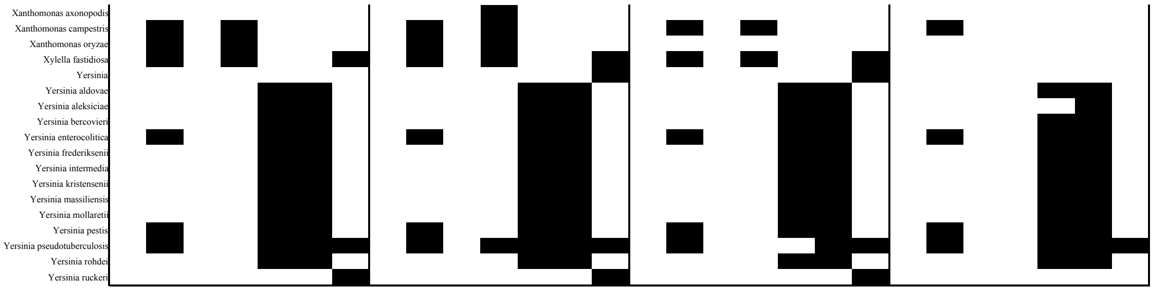

Xanthomonas axonopodis
Xanthomonas campestris
Xanthomonas oryzae
Xylella fastidiosa
Yersinia
Yersinia aldovae
Yersinia aleksiciae
Yersinia bercovieri
Yersinia enterocolitica
Yersinia frederiksenii
Yersinia intermedia
Yersinia kristensenii
Yersinia massiliensis
Yersinia mollaretii
Yersinia pestis
Yersinia pseudotuberculosis
Yersinia rohdei
Yersinia ruckeri
